# Nontarget screening of a Siberian ice core reveals changes in the pre-industrial to industrial organic aerosol composition

**DOI:** 10.1126/sciadv.adr1923

**Published:** 2025-01-24

**Authors:** François Burgay, Daniil Salionov, Thomas Singer, Anja Eichler, Sabina Brütsch, Theo M. Jenk, Alexander L. Vogel, Tatyana Papina, Saša Bjelić, Margit Schwikowski

**Affiliations:** ^1^ PSI Center for Energy and Environmental Sciences, Paul Scherrer Institute, 5232 Villigen, Switzerland.; ^2^ Oeschger Centre for Climate Change Research, University of Bern, 3012, Bern, Switzerland.; ^3^ Department of Chemistry, Biochemistry and Pharmaceutical Sciences, University of Bern, 3012 Bern, Switzerland.; ^4^ Institute for Atmospheric and Environmental Sciences (IAU), Goethe Universität, 60438 Frankfurt am Main, Germany.; ^5^ Institute for Water and Environmental Problems, Siberian Branch of the Russian Academy of Sciences, Ulitsa Molodezhnaya, 1, Barnaul, Altai Krai, Russia, 656038.

## Abstract

Glaciers serve as natural archives for reconstructing past changes of atmospheric aerosol concentration and composition. While most ice-core studies have focused on inorganic species, organic compounds, which can constitute up to 90% of the submicrometer aerosol mass, have been largely overlooked. To our knowledge, this study presents the first nontarget screening record of secondary organic aerosol species preserved in a Belukha ice core (Siberia, Russian Federation), ranging from the pre-industrial to the industrial period (1800–1980 CE). We identified a total of 398 molecules, primarily polar and low-volatile compounds. Since the 1950s, the atmospheric aerosol composition has changed, with the appearance of organic molecules, including nitrogen-containing compounds, deriving from enhanced atmospheric reactions with anthropogenic NO*_x_*, or direct emissions. In addition, there was a significant increase in the oxygen-to-carbon ratio (+3%) and the average carbon oxidation state (+18%) of the detected molecules compared to the pre-industrial period, suggesting an increased oxidative capacity of the atmosphere.

## Teaser

Untargeted analysis of an ice core reveals that human activities changed the atmospheric organic aerosol composition.

## INTRODUCTION

Atmospheric aerosol is a pivotal player in the climate system, absorbing and scattering the incoming solar radiation (*1*) and acting as cloud condensation nuclei (*2*). Organic aerosol (OA) is a major component of atmospheric aerosol, accounting for 20 to 90% of the submicrometer aerosol mass (*3*). It can be classified into primary OA, i.e., organic compounds emitted directly in the particulate form without undergoing any chemical reactions, and secondary OA (SOA), i.e., organic compounds that undergo oxidation in the gas phase, followed by condensation or nucleation (*4*). The main SOA precursors are volatile organic compounds (VOCs), which can be either of anthropogenic origin (alkanes, aromatics, and alkenes from fossil fuel combustion, with transportation and industry being the major emission sectors) or from biogenic emissions (isoprene, monoterpenes, and sesquiterpenes from terrestrial vegetation) (*5*, *6*). Once emitted into the atmosphere, VOCs are transformed to SOA reacting primarily with ^∙^OH and NO_3_^•^ radicals and O_3_ (*7*). These reactions increase the polarity of the compounds, decrease their volatility and increase their hygroscopicity, favoring their condensation on available particles (*3*). To better understand emission sources and strengths, OA requires a comprehensive chemical characterization.

While the inorganic composition of atmospheric aerosol is usually well characterized, the molecular composition of the organic fraction is challenging to reveal due to the heterogeneity in chemical properties of SOA and the large number of OA compounds, estimated to be between 10,000 and 100,000 (*8*). To overcome these difficulties, several complementary mass spectrometry techniques have been applied. For example, aerosol mass spectrometry (AMS) characterizes most of the OA mass, although it struggles to provide the molecular formula of the compounds due to extensive molecular fragmentation (*9*). Because of the polar and water-soluble nature of most biogenic SOA, liquid chromatography (LC) coupled with high-resolution mass spectrometry (HRMS) and equipped with soft ionization sources that do not result in molecular fragmentation [e.g., electrospray ionization (ESI)] has emerged as a powerful technique. Together with the development of nontarget screening (NTS) workflows, it provides an accurate mass characterization of OA species, overcoming the limitations of AMS, although at the expense of a less complete characterization of the total OA burden (*10*, *11*). While several NTS studies of the modern atmospheric aerosols exist (*12*, *13*), only a few focused on the characterization of past OA through the analysis of ice cores (*14*–*16*). To the best of our knowledge, no continuous NTS ice-core records aimed at achieving a comprehensive molecular characterization of OA over centennial time scales have been reported yet. The available long-term ice-core reconstructions of past atmospheric aerosol composition have mainly focused on inorganic species (*17*–*19*), total organic carbon (*20*, *21*), the targeted characterization of organic biomass burning tracers (*22*, *23*), specific persistent organic pollutants (*24*, *25*), and selected SOA tracers (*26*), leaving a large organic fraction unknown and uncharacterized. The limited understanding of the OA chemical composition acts as a barrier for the understanding and quantification of SOA sources and atmospheric ageing, thereby limiting our insights into past atmospheric OA composition and, consequently, on how it has changed during the industrial period. A broader and more comprehensive characterization of the OA components may also open opportunities to reconstruct the atmospheric oxidative capacity over the past centuries, thereby helping to better understand any changes in the oxidation state of trace organic compounds in the atmosphere (*27*) and the potential influence of human activities. Assessing the oxidative capacity of the atmosphere is essential to quantify the Earth’s radiative forcing as it influences the atmospheric lifetime of methane (*28*) and the hygroscopicity of OA molecules, i.e., their tendency to absorb water vapor and consequently act as cloud condensation nuclei (*3*). However, it is not trivial to quantify. A large intermodel comparison revealed uncertainties in the magnitude of ^∙^OH concentration changes from pre-industrial to present day, ranging from a decrease of 12.7% to an increase of 15.6%, with regional differences (*28*, *29*). This uncertainty is at least partly related to the difficulties in reconstructing the past atmospheric oxidative capacity from paleoenvironmental archives, such as ice cores. Attempts have been made by analyzing hydrogen peroxide (*30*) or the formaldehyde-to-methane ratio in ice cores (*31*), with results that are highly debated (*27*).

**Fig. 1.**
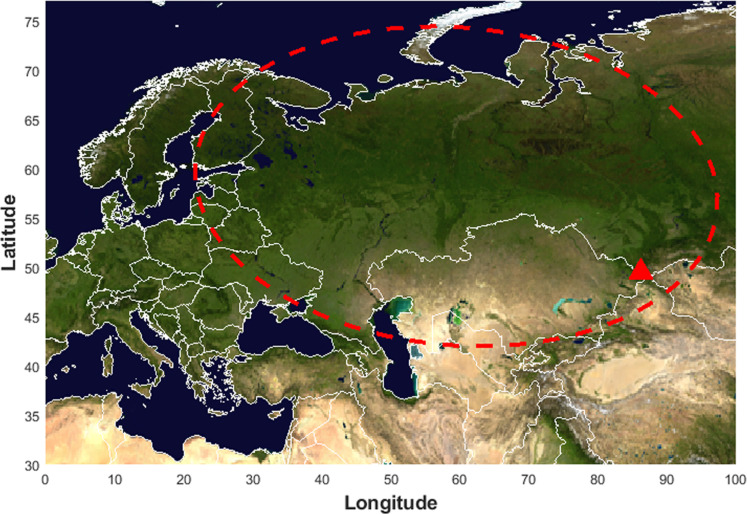
Sampling site and air-mass source regions. The location of the Belukha glacier is highlighted with a red triangle. The air mass source region, with a 7-day back-trajectory frequency > 0.1%, is qualitatively represented by the red ellipse. More details are provided in Eichler *et al.* (*33*). Mapped with Climate Data Toolbox for MATLAB (*79*).

To overcome these knowledge gaps, the first continuous NTS ice-core record unveiling the molecular identity of the OA water-soluble fraction from the Belukha glacier (Fig. 1, Russian Federation) is presented, covering the pre-industrial and industrial period (1800–1980 CE). Previous ice-core studies from a Belukha ice core have already shown that the onset of industrialization in Eastern Europe and Siberia between 1940 and 1950 CE has changed the atmospheric aerosol composition. Concentrations of major inorganic atmospheric aerosol constituents such as ammonium, nitrate, and sulfate, as well as trace elements such as Hg, Cu, Zn, Cd, Sb, and Pb increased remarkably (*32*–*34*). At the same time, the ice core reflected the strong biogenic emissions from the surrounding Taiga Belt (*35*–*37*). Thus, this glacier enables the investigation of the OA composition in the pristine, pre-industrial atmosphere, and how anthropogenic emissions have altered its chemical composition.

## RESULTS

**Fig. 2.**
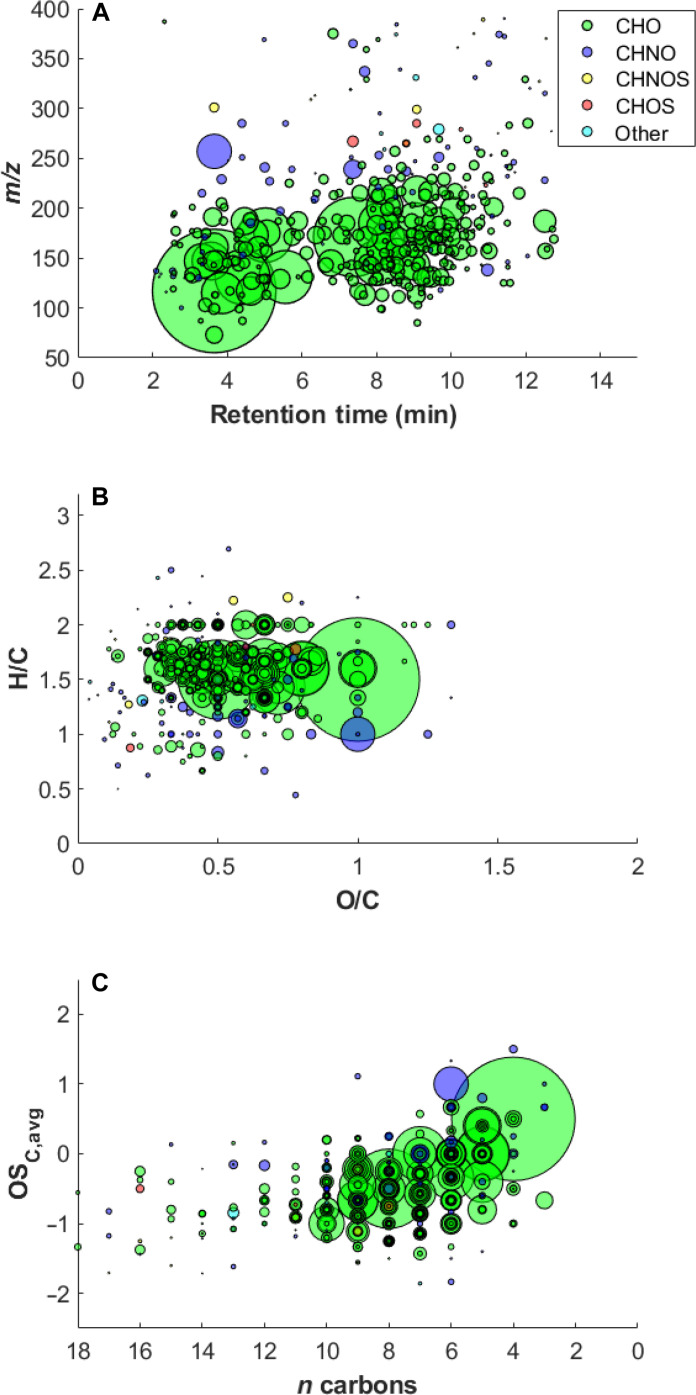
Molecular fingerprint of the compounds found in the Belukha ice core. (**A**) Mass-to-charge ratio (*m*/*z*) against RT for the 398 profiles identified after filtering (see Material and Methods for more details). (**B**) Van-Krevelen diagram. (**C**) Kroll diagram. Colors refer to the molecular class (see legend). The size of the circles is proportional to the average intensity of the specific molecules calculated over the 53 samples.

A total of 398 different molecules were identified in the Belukha ice core: 75% consist of carbon (C), hydrogen (H), and oxygen (O); 18% also contain nitrogen (N, i.e., CHNO); 3% also sulfur (S, i.e., CHNOS); 2% containing only C, O, H, and S; and 2% show presence of other heteroatoms (e.g., Cl and P), or other atomic combinations (e.g., CHN and CHNS) (hereafter defined as other) (Fig. 2A). CHO compounds account for up to 95% of the total intensity averaged over the entire record (*n* = 53 samples). This is explained both by their larger occurrence, and by the methodological setup, which was designed to enhance their ionization efficiency (*38*). The remaining 5% is divided among CHNO (4%), CHNOS (0.4%), CHOS (0.3%), and other molecules (0.3%).

The Van Krevelen diagram (Fig. 2B) shows that most of the compounds have a H/C ≥ 1.5 (*n* = 247), implying that the majority of the molecules is aliphatic (fig. S1) (*39*) with double bond equivalent (DBE; see Materials and Methods for more details) values less than 4 (*n* = 260) (fig. S2). These findings are further supported by the aromaticity equivalent (χ_c_, see Materials and Methods for more details), which is a conservative tool to assess the identification of aromatic compounds (*40*). χ_c_ ≥ 2.5 is the threshold that is set to identify the presence of aromatics. In our dataset, 17% (*n* = 66) of the compounds have χ_c_ ≥ 2.5, confirming the aliphatic nature of most of the molecules identified in the Belukha ice core (figs. S3 and S4). We should caution that the use of this parameter, while useful and informative, might still underrepresent the actual number of aromatic molecules. For example, C_8_H_8_O_3_ has a χ_c_ = 2.4, while potentially referring to compounds with an aromatic structure [p-(hydroxymethyl) benzoic acid, or vanillin]. Last, 76% of compounds (*n* = 306) has 0.25 ≤ O/C < 0.75, indicating the presence of molecules, deriving, for example, from the oxidation of isoprene or monoterpenes (*41*, *42*) (fig. S1).

The Kroll diagram (Fig. 2C) shows the average carbon oxidation state (OS_C,avg_ = 2 O/C − H/C) of the compounds versus their number of carbon atoms (nC) (*43*). For the large majority of the compounds (*n* = 330) the OS_C,avg_ is ≥ −1 (fig. S1), indicating that they can be products of monoterpenes ozonolysis or of the reaction between monoterpenes and ^∙^OH (−1 ≤ OS_C,avg_ ≤ −0.5), products of reaction between isoprene and ^∙^OH (−0.8 ≤ OS_C,avg_ ≤ −0.2), semi-volatile oxygenated compounds (SVOCs, −0.5 ≤ OS_C,avg_ ≤ 0), or low-volatile oxygenated compounds (LVOCs, 0 ≤ OS_C,avg_ ≤ +0.9) (*44*, *45*). The low occurrence of compounds with −1.7 ≤ OS_C,avg_ ≤ −1.6 (*n* = 8) indicates either the low abundance of hydrocarbon-like OA in the samples or is a consequence of the analytical methodology, which detects water-soluble compounds (*38*).

### Molecular classes

Eighty percent of CHO compounds have a DBE < 4, and 95% an χ_c_ < 2.5, indicating that they are aliphatic, with the majority (62%) having between 2 and 3 double bonds. Considering that 87% of them have an OS_C,avg_ higher than −1, and that 90% appear in the range 0.3 ≤ O/C ≤ 1 (fig. S4), CHO compounds can be associated with aliphatic water-soluble SOA compounds, deriving from the atmospheric oxidation of monoterpenes and isoprene (*14*, *43*). Since most of the molecules contain between 2 and 5 oxygen atoms (83%), they can be esters, glycols, di-ketones, mono-, or di-carboxylic acids (fig. S4). Their identity has been partly revealed by the detection of several carboxylic acids at different identification confidence levels (table S1).

**Fig. 3.**
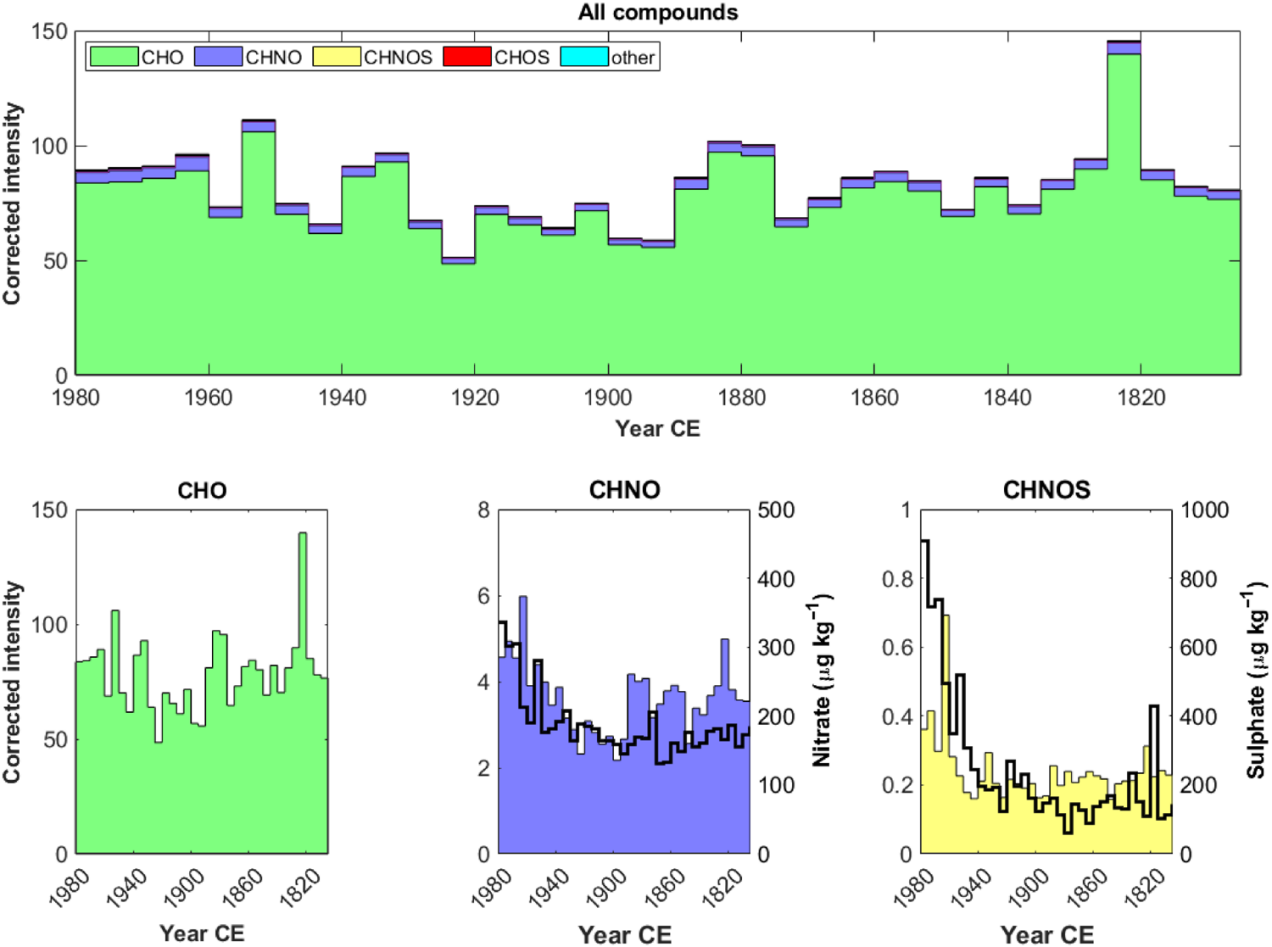
Temporal records of the different compound classes identified in the Belukha ice core from 1800 CE to 1980 CE. CHOS and other are not shown because of their low contribution and absence of any temporal trend. The concentrations of nitrate and sulfate are also reported (black lines).

The identification confidence levels refer to the Schymanski’s scale (*46*) and they range from level 5 (i.e., the lowest level of identification, where only the mass-to-charge ratio is known) to level 1 (i.e., the highest level of identification, where the identity of the compounds is confirmed with a reference standard). The intermediate levels refer to: (i) compounds whose molecular formulas have been computed (level 4), (ii) the identification of tentative candidates with similar structures (level 3), and (iii) compounds that resulted in unambiguous matches with available spectral libraries (level 2). In the ice-core samples, different homolog series have been identified at different confidence level of identifications. A dicarboxylic homolog series (C*_n_*H_2*n*−2_O_4_, *n* = 4, 5, 6, 7, 8, 9, and 10) including succinic acid [level 1, C_4_H_6_O_4_, retention time (RT) = 3.68 min], glutaric acid (level 1, C_5_H_8_O_4_, RT = 4.44), adipic acid (level 1, C_6_H_10_O_4_, RT = 6.41 min), pimelic acid (level 1, C_7_H_12_O_4_, RT = 8.17 min), and sebacic acid (level 1, C_10_H_18_O_4_, RT = 11.13 min) was identified. Also, pinic acid (level 1, C_9_H_14_O_4_, RT = 9.03 min) and its homologs (C*_n_*H_2*n*−4_O_4_, *n* = 5, 6, 7, 9, and 10) were observed. Other homolog series referred to: (i) hydroxy fatty acids (C*_n_*H_2*n*_O_3_, *n* = 4, 5, 6, 7, and 8), including hydroxybutyric acid (level 3, C_4_H_8_O_3_, RT = 3.41 min, mzCloud match = 86.5); (ii) unsaturated monocarboxylic acids (C*_n_*H_2*n*−2_O_2_, *n* = 4, 5, 7, 8, and 9), including crotonic acid (level 2, C_4_H_6_O_2_, RT = 9.09 min, mzCloud match = 79.5); (iii) hydroxy dicarboxylic acids (C*_n_*H_2*n*−2_O_5_, *n* = 5, 6, 7, 8, and 9), including 3-hydroxyglutaric acid (level 2, C_5_H_8_O_5_, RT = 3.57 min, mzCloud match = 68.8), 3-hydroxy-3-methylglutaric acid (level 2, C_6_H_10_O_5_, RT = 3.65 min, mzCloud match = 87.2), and isopropylmalic acid (level 3, C_7_H_12_O_5_, RT = 4.31 min, mzCloud match = 84.8); (iv) monocarboxylic acids (C*_n_*H_2*n*_O_2_, *n* = 4, 5, 6, 7, and 8) including heptanoic acid (level 2, C_7_H_14_O_2_, RT = 9.64 min, mzcCloud match = 67.0); and (v) ketoacids (C*_n_*H_2*n*−2_O_3_, *n* = 5, 6, 7, 8, 9, and 10) including levulinic acid (level 1, C_5_H_8_O_3_, RT = 3.87 min). Other identified compounds were aromatic carboxylic acids, some of them tracers for biomass burning (*47*) such as p-hydroxybenzoic acid (level 1, C_7_H_6_O_3_, RT = 8.55 min) and p-hydroxybenzaldehyde (level 2, C_7_H_6_O_2_, RT = 8.88 min, mzCloud match = 86.9); caffeic acid, p-hydroxyphenylpyruvic acid, or 2-3-dihydro-1,4-benzodiozine-5-carboxylic acid (level 3, C_9_H_8_O_4_, RT = 8.37 min); and 3-methylsalicylic acid, 4-hydroxy-2-methylbenzoic acid, or p-hydroxyphenylacetic acid (level 3, C_8_H_8_O_3_, RT = 9.83 min). The aggregated CHO profile record has an overall low variability over the entire period [coefficient of variability (CV) = 0.20], and it does not show any significant trend (*P* value = 0.4) (Fig. 3). The highest intensity is observed between 1820 and 1825.

CHNO compounds are the second most abundant molecular class in the ice-core record, contributing up to 4% of the overall intensity. Their high DBE values (75% of them having DBE ≥ 4), low H/C values (66% of them having H/C < 1.5), and a high number of compounds with χ_c_ ≥ 2.5 (54%), indicate a higher degree of unsaturation and aromaticity compared to the other classes (*14*, *48*). Their high O/C ratio (54% has O/C ≥ 0.5) indicates that they have a high oxidation state (fig. S4). At level 2, p-nitrophenol (C_6_H_5_NO_3_, RT = 10.94 min, mzCloud Match = 92.7), which is formed in a NO*_x_*-rich atmosphere (*49*), such as during forest fires (*14*) or during fossil fuel combustion (*50*), was identified together with 5-aminolevulinic acid (C_5_H_9_NO_3_, RT = 3.20 min, mzCloud Match = 77.8), 5-aminovaleric acid (C_5_H_11_NO_2_, RT = 2.37 min, mzCloud Match = 65.6), and 3-methylhippuric acid (C_10_H_11_NO_3_, RT = 11.10 min, mzCloud Match = 60.2). At level 3, nine molecules were identified, including C_6_H_5_NO_3_ (6-hydroxypicolinic acid or hydroxynicotinic acid), C_6_H_6_N_2_O_2_ (aminonicotinic acid or 4-nitroaniline), C_6_H_11_NO_3_ (4-acetamidobutanoic acid or *N*-isobutyrylglicine), and C_7_H_7_NO_3_ (aminosalicylic acid or mesalamine). At level 4 (i.e., when only the molecular formula is provided), several compounds identified at the same level in modern atmospheric aerosol samples were observed, such as: C_7_H_7_NO_3_ (RT = 12.05 min) (*51*), C_19_H_37_NO_6_ (RT = 11.28 min) (*52*), C_6_H_6_N_2_O_2_ (RT = 2.10 min) (*53*), C_4_H_7_NO_4_ (RT = 2.56 min) (*52*), C_11_H_19_NO_3_ (RT = 10.58) (*54*), and C_5_H_9_NO_3_ (RT = 3.23 min) (*55*). The aggregated CHNO record shows relatively high intensities between 1800 and 1945, followed by a 1.4 fold increase in 1950. In fig. S5, we present some of the CHNO temporal profiles, which have different onset years and trends.

CHNOS compounds contribute just 0.4% to the overall intensity, consistent with their limited occurrence (*n* = 11) in the record. Compared to CHO and CHNO, CHNOS compounds are less oxidized (82% have O/C < 0.5). In addition, their H/C ratio values peaking at 1.75 ≤ H/C < 2, indicate that most of these compounds have an aliphatic structure (fig. S4), as also indicated by the χ_c_ < 2.5 for 60% of the molecules. It was not possible to identify any CHNOS compound at higher identification confidence level than level 4. Like CHNO, the aggregated CHNOS profile shows an abrupt change point in 1960 CE, with a 1.9-fold increase compared to the 1800–1955 period, indicating that anthropogenic activities have enriched the atmospheric aerosol also in CHNOS molecules (Fig. 3). In fig. S6, some of the CHNOS temporal profiles are presented, with their different onset years and trends. CHOS and other compounds will not be discussed further due to their low occurrence and low contribution to the overall intensity.

### Hierarchical cluster analysis

**Fig. 4.**
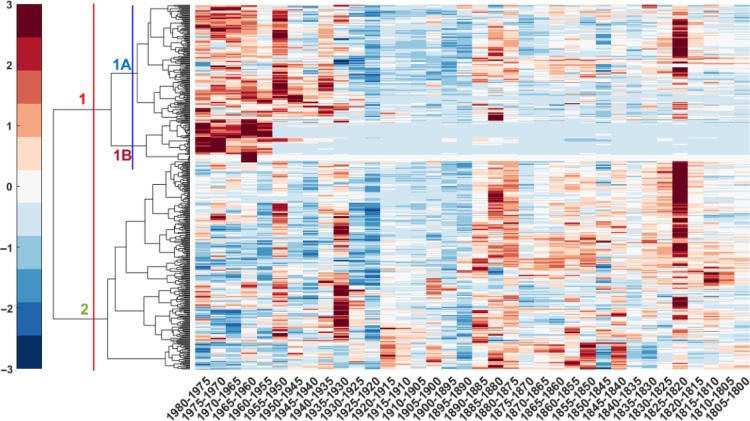
HCA of scaled data against time (year CE). *X* axis represents the different years (5-year average). *Y* axis represents the compounds. Colors refer to the molecules’ *z* score ranging from −3 (dark blue) to +3 (dark red). Two clusters were identified, defined as anthropogenic (1) and natural (2) clusters respectively. Cluster 1 was further divided into two subclusters (1A and 1B).

To identify relationships between the 398 compounds, a hierarchical cluster analysis (HCA) was conducted (Fig. 4), resulting in two main clusters, namely, 1 and 2. Cluster 1 was further divided into two subclusters, namely, 1A and 1B.

#### Cluster 1 – Anthropogenic cluster

**Fig. 5.**
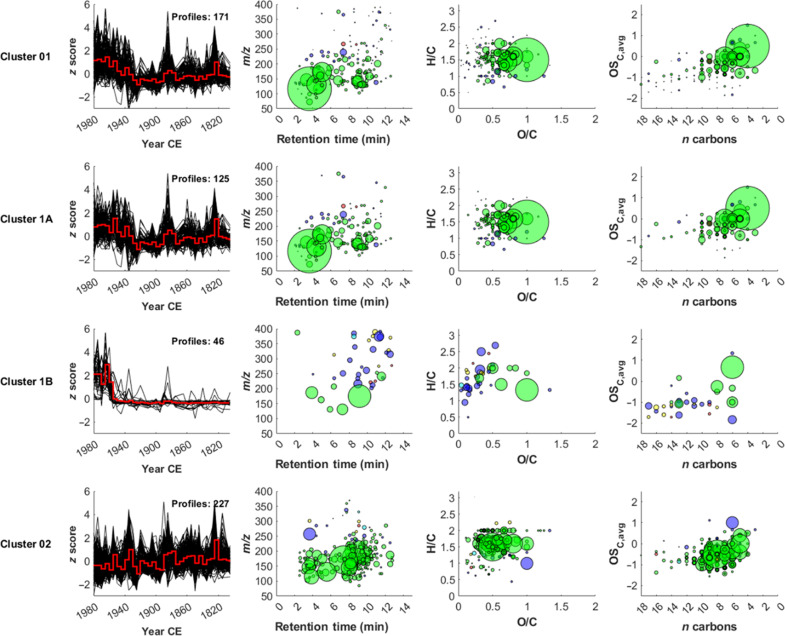
Temporal records (5-year averages) and molecular characterization (RT, O/C, and nC) of the different clusters. For each cluster, the number of molecules (i.e., profiles) is also reported. The size of the circles in cluster 1B is magnified by 10 to account for the low intensities of the molecules associated with it. Colors refer to the molecular classes (green: CHO, purple: CHNO, yellow: CHNOS, red: CHOS, and cyan: other).

Compounds identified in cluster 1 (*n* = 171) were attributed to anthropogenic emissions since the cluster mean (*z* score) shows an abrupt change point in 1950 CE and is correlated with concentrations of NO_3_^−^ (*r* = 0.70) and SO_4_^2−^ (*r* = 0.72), which are proxies for fossil fuel combustion (*56*), and with different heavy metals (e.g., Cu, Zn, Cd, Sb, and Pb) that also relate to anthropogenic activities and emissions (e.g., nonferrous metal production, road traffic) (table S2). Therefore, this cluster is named anthropogenic cluster. Of the two subclusters, cluster 1A includes molecules that must also have a biogenic source, since they were present even before the onset of industrialization, around 1940 CE in Eastern Europe (*n* = 125), while cluster 1B includes molecules that have a purely anthropogenic source being entirely absent in the pre-industrial period (*n* = 46). Cluster 1A predominantly consists of CHO compounds (*n* = 93), followed by CHNO (*n* = 27), CHOS (*n* = 3), and other molecules (*n* = 2). The ensemble mean (*z* score) of this cluster (Fig. 5) shows no significant trend before 1925 CE (*P* value = 0.06), but a significant increase between 1925 and 1980 CE (*P* value = 0.02). The average O/C is 0.6 ± 0.2 and the average oxidation state of carbon is −0.3 ± 0.5, indicating that the molecules belonging to this cluster are highly oxidized and mainly composed of semivolatile oxygenated organic compounds (*14*). The average DBE (3 ± 2) suggests that most of the compounds have a low number of unsaturation (fig. S2), consistent with a low χ_c_ (1 ± 1) that hints to their aliphatic nature (fig. S3). Among the most intense compounds identified at the highest confidence level, aliphatic di-carboxylic acids such as succinic acid (C_4_H_6_O_4_), adipic acid (C_6_H_10_O_4_), sebacic acid (C_10_H_18_O_4_), and glutaric acid (C_5_H_8_O_4_) were observed. These compounds, while having a biogenic source, also derive from the atmospheric oxidation of anthropogenic VOCs (*57*). Biomass burning tracers such as p-hydroxybenzoic acid (C_7_H_6_O_3_) and p-hydroxybenzaldehyde (C_7_H_6_O_2_) were also identified together with other organic molecules having both a biomass burning and an anthropogenic source like p-nitrophenol (C_6_H_5_NO_3_) and C_7_H_7_NO_3_, which was tentatively attributed to methyl-nitrophenol considering its high correlation with p-nitrophenol (*r* = 0.91). In addition, in this cluster, at least three main homolog series were observed, which were tentatively attributed to saturated and unsaturated dicarboxylic acids and hydroxy-dicarboxylic acids.

Cluster 1B is largely composed of CHNO compounds (*n* = 21), followed by CHO (*n* = 9), CHNOS (*n* = 7), CHOS (*n* = 3), and other molecules (*n* = 6). The average O/C is 0.4 ± 0.3 and the average oxidation state of carbon is −1 ± 0.6, indicating relatively low oxidized molecules. The higher DBE (4 ± 3) and χ_c_ (1.6 ± 1.2) values suggest more unsaturated and aromatic compounds than cluster 1A (fig. S3). Even though no matches were found between the acquired fragmentation spectra (MS^2^) and the consulted online spectral libraries, the results highlight the occurrence of previously unknown substances, which may be directly or indirectly related to emission into the atmosphere from the nearby industries (*32*). C_21_H_20_N_2_O_2_, C_19_H_37_NO_6_, C_13_H_35_N_5_O_7_, and C_8_H_12_O_5_ were detected among the most intense profiles.

#### Cluster 2 – Natural clusters

Cluster 2 includes most of the molecules observed in the Belukha ice core (*n* = 227 of 398). As they were present throughout the record and do not correlate with either NO_3_^−^ or SO_4_^2−^, this cluster is named natural cluster*.* The dominant molecular class is CHO (*n* = 195), followed by CHNO (*n* = 24), CHNOS (*n* = 4), CHOS (*n* = 2), and other compounds (*n* = 2). The ensemble mean (*z* score) of this cluster (Fig. 5) shows a long-term decreasing trend (*P* value = 0.01) between the 19th and 20th century. The average O/C for cluster 2 is 0.5 ± 0.2, and the average carbon oxidation state is −0.6 ± 0.5, indicating that, although this cluster is composed of SOA tracers derived from the oxidation of monoterpenes and isoprene with a similar degree of unsaturation (DBE = 3 ± 2; fig. S2) and aromaticity (0.9 ± 0.9) as cluster 1A (fig. S3), it contains fewer oxidized molecules (fig. S1). A *t* test performed on both the O/C (*P* value = 0.001) and the average carbon oxidation state (*P* value = 5 × 10^−5^) for molecules belonging to clusters 1A and 2 supports this interpretation. Among the most intense compounds, pimelic acid (C_7_H_12_O_4_) and levulinic acid (C_5_H_8_O_3_) were observed. In addition, in this cluster, at least 12 homolog series were found. According to their higher number of occurrences, we tentatively attribute some of them to hydroxy fatty acids (C*_n_*H_2*n*_O_3_) and ketoacids (C*_n_*H_2*n*−2_O_3_).

## DISCUSSION

### Changes in the atmospheric aerosol composition

In this study, 177 different compounds that have been directly or indirectly affected by anthropogenic perturbation during industrialization, i.e., those belonging to cluster 1, were observed. Among them, 46 (i.e., cluster 1B) were observed only in the most recent part of the core, indicating an abrupt change in the atmospheric OA composition, with larger occurrences of CHNO and CHNOS compounds (Fig. 5). These molecules were either directly emitted or formed in the atmosphere by oxidation of other precursors. The larger occurrence of CHNO and CHNOS molecules suggests atmospheric oxidative pathways involving anthropogenic NO*_x_* and SO_2_. While nitrate chemistry was active even before the onset of industrialization, as shown by the high CHNO and nitrate background values (Fig. 3), the overall intensity of CHNO compounds was higher in the industrial period likely caused by stronger atmospheric NO*_x_* emissions (*37*, *58*). At low NO*_x_* concentrations, peroxy radicals (RO_2_^•^), which are formed after the initial oxidation step, then typically undergo reactions with hydroperoxy radicals (HO_2_^•^) to form organic hydroperoxides (ROOH). RO_2_^•^ can also react with itself eventually generating alcohol and carbonyl products or form dimers by recombination with other RO_2_^•^. In contrast, at high NO*_x_* concentrations, RO_2_^•^ primarily reacts with nitric oxide (NO) to form either organonitrates (RONO_2_) or alkoxy radicals (RO^•^) and nitrogen dioxide (NO_2_). RONO_2_ can eventually be deposited or lost by oxidation, photolysis, and hydrolysis (*59*). RO_2_^•^ can also react with NO_2_ producing peroxy-nitrate (RO_2_NO_2_). Even though peroxy-nitrates can have longer atmospheric lifetimes at low temperatures (i.e., typical of those at high elevations), this is not a major pathway (*7*, *60*). A third mechanism to produce organonitrates involves the reaction between biogenic VOC (BVOC) and NO_3_ radicals by NO_3_ addition to the double bonds and aromatic rings. Even though NO_3_^•^ chemistry is mainly active in the nighttime atmosphere due to the rapid NO_3_^•^ photolysis in sunlight, BVOCs are particularly susceptible to oxidation by NO_3_^•^ due to the presence of double bonds in their structure. In the ice-core record, at least two molecular formulas (C_10_H_15_NO_7_, RT = 10.69 min; and C_10_H_13_NO_6_, RT = 11.48 min) compatible with organonitrates deriving from NO_3_^•^ + β-pinene oxidation were observed, suggesting the occurrence of this reaction mechanism and its enhancement during the industrial period (*61*).

The change in the emission mix also led to a small, yet significant, increase from 2.62 ± 0.02 (1800–1950 CE) to 2.66 ± 0.04 (1950–1980 CE) of the weighted averaged DBE values (fig. S2, first panel), while no significant trend was observed for aromaticity (fig. S3, first panel). Even though aliphatic compounds with two and three double bonds, such as dicarboxylic acids, still explain most of the intensity during both the pre-industrial and industrial periods, the anthropogenic cluster contains slightly more unsaturated compounds justifying the increase in the DBE from 1950 CE. It should be considered that, while the DBE increase was small, the molecular intensities used to calculate these parameters do not reflect the actual concentration of the compounds, but rather their ionization efficiencies, which in turn depend on their molecular structure and on the methodological setup.

### Changes in the average molecular oxidation states

**Fig. 6.**
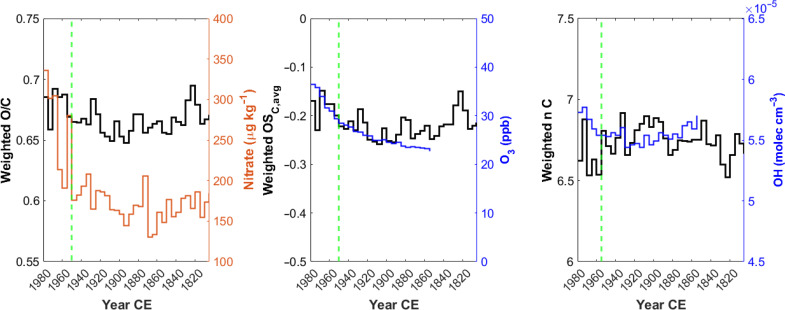
Changes in the molecular oxidation state. (**Left**) 5-year averaged record of the weighted oxygen-to-carbon ratio (black line) and nitrate concentration (orange line). (**Middle**) 5-year averaged record of the weighted carbon oxidation state (black line), and ozone mixing ratio in the atmospheric surface layer (blue line). (**Right**) 5-year averaged record of the weighted number of carbon atoms (nC, black line) and ^∙^OH concentration (blue line). The dotted green vertical lines represent the abrupt change points (1950 CE or 1955 CE for the weighted number of carbons).

To assess changes in the oxidation state of organic compounds, the contribution of molecules with a purely anthropogenic source (i.e., cluster 1B) was neglected and the focus was placed only on molecules also having a biogenic source (i.e., clusters 1A and 2). This choice was made to avoid any possible influence of compounds emitted primarily from anthropogenic sources that do not experience any atmospheric oxidation. Only considering clusters 1A and 2, relatively constant weighted average values for O/C and OS_C,avg_ were observed before 1950 CE (0.66 ± 0.01 and −0.22 ± 0.02, respectively) and for the weighted average nC values before 1955 (6.75 ± 0.09). In 1950 CE (1955 CE for nC), a significant increases up to 0.68 ± 0.01 (+3%) and −0.18 ± 0.03 (+18%) were observed for O/C and OS_C,avg_, respectively, and a decrease of 6.6 ± 0.1 (−2%) for nC (Fig. 6).

The increase in the oxidation state of the compounds is consistent with the change in the relative intensities between cluster 1A and cluster 2 (fig. S7). As discussed above, cluster 1A is characterized by more oxidized compounds, on average, than cluster 2 (fig. S1), and its relative intensity increased from 41 ± 3% in the period 1800–1950, to 49 ± 3%, between 1950 and 1980, while that of cluster 2 decreased. To explain the change in the overall molecular oxidation state, three hypotheses are advanced. The first hypothesis relies on the fact that anthropogenic activities have increased the concentration of atmospheric oxidants (e.g., O_3_, NO_3_^•^, and ^•^OH), leading to the formation of more oxidized compounds, i.e., increasing the oxidative capacity of the atmosphere. This hypothesis is supported by progressively higher ozone mixing ratio concentrations in the Belukha source region during the industrial period (fig. S8), with an abrupt change point observed in 1950 CE (Fig. 6). The ozone mixing ratio calculated in the atmospheric surface layer is significantly correlated with the OS_C,avg_ (*r* = 0.66), and its 28% increase between 1850–1950 and 1950–1980 is comparable to that observed for the OS_C,avg_. In addition, the O_3_ concentration temporal profile significantly correlates with cluster 1A (*z* score, *r* = 0.66), therefore suggesting a direct link between progressively higher tropospheric ozone concentrations and the occurrence of more oxidized compounds. These results are also consistent with previous studies that showed an increase of ozone concentration in the industrial period compared to the pre-industrial from the Belukha source areas (*29*). However, O_3_ plays only a minor role in oxidizing organic compounds in the atmosphere, with hydroxyl radicals (^∙^OH) playing the primary role in this process. An increase in atmospheric ^∙^OH concentrations would likely enhance the oxidation state of these compounds while also reducing the nC in SOAs due to fragmentation, a key process in the oxidative aging of atmospheric organics (*43*). In the Belukha record, a 2% decrease in the weighted average nC was observed between 1955 and 1980 CE, alongside a small increase in ^∙^OH concentrations (+1%) compared to the previous period (1980–1950 CE). The decrease in the nC is consistent with the increasing contribution of cluster 1A to the overall intensity (fig. S7), as it contains compounds with a significantly lower nC (*P* value = 0.008) than cluster 2 (fig. S1). However, the observed changes in nC and modeled ^∙^OH concentrations were not statistically significant compared to the average calculated over the period 1800–1950 CE (*P* value = 0.08 and 0.40, respectively). This finding suggests that, while ^∙^OH concentration shows a significant increasing trend since the 1930s CE (*P* value = 8 × 10^−4^), this increase was not sufficient to enhance organic fragmentation compared to the pre-industrial period. Despite considerable uncertainties in calculating ^∙^OH, primarily due to challenges in modeling factors that can enhance (e.g., humidity, tropospheric ozone, NO*_x_* emissions, and ultraviolet radiation) or reduce (e.g., methane concentration and carbon monoxide emissions) its concentrations, our findings are consistent with large intermodel comparisons, which indicate little changes in atmospheric ^∙^OH since the beginning of the industrial period, including the Belukha source region (*28*, *29*, *62*). Also, the small and statistically insignificant decrease in nC could be seen as an independent assessment of the limited changes in ^∙^OH concentration compared to the pre-industrial period in this region, which have not enhanced molecular fragmentation. Overall, while ^∙^OH contribution seems to be relatively small and unchanged over the last 180 years, the primary driver of the increased oxidation state of these compounds appears to be tropospheric O_3_, which showed the strongest variability and whose recent increase might have driven an enhancement in the molecular oxidation state.

Nevertheless, other processes may have also played a role in explaining the higher oxidation state of compounds in the industrial period. This brings to the second hypothesis that relies on the presence of more acidic aerosols, enhanced by the emissions of NO*_x_* and SO_2_ that are oxidized in the atmosphere to HNO_3_ and H_2_SO_4_. More acidic aerosol conditions could have acid-catalyzed SOA formation from isoprene or monoterpenes, accelerating its rate compared to neutral conditions and leading to more oxidized end products (*63*). The third hypothesis relies on the progressively higher nonmethane volatile organic carbon (NMVOC) emissions derived from fossil sources in the Former Soviet Union that significantly increased between 1950 and 1980 CE (*64*). The atmospheric oxidation of anthropogenic NMVOC might have led to the formation of highly oxidized compounds in addition to those formed from the oxidation of BVOC, explaining the observed trend in cluster 1A. This is supported by the increasing trend observed for some highly oxygenated dicarboxylic acids (e.g., succinic acid) that also derive from the atmospheric oxidation of anthropogenic VOCs (*57*). Overall, all these hypotheses imply that anthropogenic pollution in the Former Soviet Union has altered the oxidation state of OA, leading to the formation of more oxidized compounds. The change in the oxidation of the water-soluble organic compounds influences their hygroscopicity; therefore, their capacity to act as cloud condensation nuclei, with potential effects on the Earth’s radiative forcing.

An increase in the CHO intensities and in the O/C and OS_C,avg_ and a decrease in the nC were also observed between 1820 and 1825 CE. This may be related to the Tambora volcanic eruption in 1815 CE, as confirmed by the large increase in sulfate concentration (Fig. 2). Tambora was the largest volcanic eruption of the last millennia, causing a global increase in atmospheric aerosol acidity lasting up to 3 years in the Northern Hemisphere (*65*) and a global cooling and droughts that lasted up to 4 years after the eruption (*66*). The prolonged acidity increase may have enhanced SOA mass concentration and atmospheric processing of SOA precursors (*67*), and/or second, vegetation may have increased VOC emissions after reduced vegetation growth caused by eruption related solar dimming and induced drought stress (rebound effect) (*68*). An additional explanation may also come from increased water vapor concentrations due to volcanic eruptions, resulting in elevated concentration of OH radicals (*69*). Higher ^∙^OH concentrations might have decreased the average nC due to fragmentation and, contextually, increased their oxidation. Further studies are required to assess the influence of volcanic eruptions on the OA composition.

### Limitations

This study provides the first NTS ice-core record that aimed at reconstructing changes in the atmospheric OA composition from the pre-industrial to the industrial period. Our results show that the atmospheric aerosol composition and the average oxidation state of organic compounds has changed over the last two centuries. Even though an unprecedented molecular characterization of the organic compounds present in ice cores has been achieved, there are limitations that need to be addressed in future investigations: (i) The low enrichment factors used to preconcentrate the samples (92 ± 3) limit the assessment of the anthropogenic impact on the atmospheric aerosol composition since the low concentrations of organic pollutants in the ice typically require higher enrichment factors (≥500) (*25*, *70*, *71*). Future NTS studies are encouraged to use larger ice volumes; (ii) in this study, three main molecular classes have been described. These classes do not represent the entire OA composition, which has been constrained by both the choice of strong anionic exchange solid-phase extraction cartridges in the pre-analytical step and by the (−)ESI ionization mode during the analysis. This methodological setup did not allow the effective preconcentration of small and neutral molecules as well as the ionization of nonpolar compounds and molecules without acidic protons. Furthermore, although organosulfates can account for up to 30% of the total OA mass fraction (*72*), the methodology used in this study did not allow the detection of a high number of CHOS species. Their detection usually requires optimized and dedicated analytical procedures and, in some cases, the use of specific ionization promoters or derivatization (*73*, *74*).

## MATERIAL AND METHODS

### Sampling site, sample processing, and labware cleaning procedure

A 163-m–long ice core reaching bedrock was collected using an electromechanical drill at Belukha glacier (4062 m above sea level, 49°48′26″ N, 86°34′46″ E; Fig. 1) between 27 May and 10 June 2018. The core was drilled around 90 m NE of the 2001 drill site (B01) (*75*). After being collected, all cores were sealed in polyethylene tubes in the field and stored in insulated boxes. All samples were shipped frozen to Paul Scherrer Institut (Switzerland) and stored at −20°C until analysis. The samples analyzed in this study (*n* = 53) cover the time period 1800–1980 CE, i.e., from to 62.87 to 26.55 m depth. Samples had a density ≥ 0.7 g cm^−3^, thus minimizing risks for external contamination that can occur when porous material, such as firn, is analyzed. More details on ice-core processing and the labware cleaning procedure are reported in section S1.

### Extraction and mass spectrometric analysis

Water-soluble organic tracers were extracted and analyzed following the methodology described in Burgay *et al.* (*38*). In brief, samples were enriched by a factor 92 ± 3 using strong anionic exchange solid-phase extractions cartridges (MAX, Waters) and analyzed using ultra-high performance liquid chromatography (Ultimate 3000, Thermo Fisher Scientific) equipped with an Acclaim Organic Acid Column (3 μm, 2.1 × 150 mm, Thermo Fisher Scientific, operated at 50°C) coupled with high-resolution mass spectrometry (UHPLC-HRMS, Q Exactive Focus, Thermo Fisher Scientific). This setup allowed an optimal ionization efficiency and chromatographic separation for the class of compounds of interest, i.e., SOA tracers, such as carboxylic acids, while it is not suitable for the complete analyses of S-containing molecules, due to their high diversity (e.g., sulfides, thiophenes, or polyaromatic sulfur heterocycles) that require other pre-analytical and analytical conditions. More details about the standards and solvents used for the extraction, analysis, and identification are provided in section S2.

### Data processing

Data were preprocessed using Compound Discoverer 3.2 (Thermo Fisher Scientific) and then treated for statistical analyses with MATLAB (MathWorks). To smooth year-to-year variability of the atmospheric transport and snow preservation, the statistical analyses and environmental interpretation were performed on 5-year averages (*56*).

#### Compound discoverer settings

The settings used for Compound Discoverer are described in detail in the supplementary information of Burgay *et al.* (*38*). Of all the profiles identified, an additional filtering was applied: (i) background is false, (ii) peak rating ≥ 7.0, (iii) group CV ≤ 15%, (iv) formula is not blank, (v) max intensity ≥ 5 × 10^6^, and (vi) intensity threshold for the sample-to-blank ratio of 3.

#### Identifications

Compounds were identified according to the Schymanski scale (*46*). To identify the molecules found in the Belukha ice core at level 2, online spectral libraries (i.e., mzCloud and the NORMAN Mass Bank) for MS^2^ spectra comparison were used (*76*). For level 1 identifications, an in-house database was used for MS^2^ spectra and RTs comparison between the suspects and the reference standards. The suspects were confirmed at level 1 when ΔRTs ≤ 0.1 min.

#### Statistical analyses

HCA was used to reduce the complexity of the dataset, to identify sample and molecular clusters, and to prioritize molecular identifications. Initially, data were standardized based on *z* transformation (zscore function), then clustered using the clustergram function with Euclidean as the metric for distance computation and the Ward method for creating the agglomerative hierarchical cluster tree. To detect abrupt change points in the time series, the findchangepts function was used. Linear correlations were performed using the corr function and in case of missing data, linear interpolation was performed using the interp1 function. To evaluate the significance of temporal trends, the fitlm function was used and the associated *P* value was evaluated for significance. The statistical significance of the tests was set to 0.05.

#### DBE and aromaticity equivalent

The DBE is defined as DBE = *C* − (*H*/2) + (*N*/2) + 1, where *C* is the nC, *H* is the number of hydrogen atoms and halogen atoms, and *N* is the number of nitrogen atoms. The aromaticity equivalent (χ_c_) is calculated as

$$\chi_{c}=\frac{3\cdot [\text{DBE}-(m\cdot \#O+n\cdot \#S)]-2}{\text{DBE}-\left(m\cdot \#O+n\cdot \#S\right)}\;\left(0\;\text{if}<0\right)$$ (1)

Where *m* and *n* are the fractions of oxygen and sulfur atoms in the π-bond structures of a compound. Usually, these parameters are tuned depending on the compound classes. For example, *m* = *n* = 0.5 for carboxylic acids and esters, while *m* = *n* = 1 and *m* = *n* = 0 for carbonyl and hydroxyl, respectively. As it is impossible to identify the structures of all the compounds of this study, *m* and *n* were set to 0.5, also considering that carboxylic acids are more efficiently ionized in the (−)ESI (*10*). χ_c_ ≥ 2.5 is the threshold that is set to identify the presence of aromatics.

### Ice-core dating

The ice core was dated using δ^18^O and NH_4_^+^ for annual layer counting, constrained with the 1963 CE nuclear fallout maximum detected by the ^3^H peak, and with nondust SO_4_^2−^ peaks attributed to volcanic eruptions (Katmai, 1912 CE; and Tambora, 1815 CE). The ice-core record presented in this work covers the period 1800–1980 CE. The upper part of the record was not analyzed to avoid external contamination since it consisted of firn and had a density below 0.7 g cm^−3^, therefore open porous to the ambient atmosphere and prone to potential contamination. The dating uncertainty varies with proximity to the surface and the three reference horizons (1963 CE, 1912 CE, and 1815 CE), with at most ±5 years between 1883 CE and the surface and ±10 years between 1815 and 1883 CE.

### Major ion analyses

Concentrations of SO_4_^2−^ and NO_3_^−^ were analyzed using ion chromatography (850 Professional IC equipped with an 872 Extension Module Liquid Handling and an 858 Professional Sample Processor autosampler, Metrohm, Herisau, Switzerland) (*77*). Their trends were compared with the corresponding records from the B01 ice core (*37*), showing a significant 5-year average correlation for both sulfate (*r* = 0.93) and nitrate (*r* = 0.88) over the period 1800–2000 CE.

### Ozone and ^∙^OH concentrations

The monthly resolved ozone mixing ratio in the atmospheric surface layer values and ^∙^OH radical concentrations between 1850 CE and 1980 CE were retrieved from the Community Earth System Model Version 2 (CESM2), available at https://earthsystemgrid.org/dataset/ucar.cgd.cesm2.output.html (*78*) and the CESM2.1 run b.e21.BWHIST.f09_g17.CMIP6-historical-WACCM.001, Atmosphere Post Processed Data, Monthly Averages, version 2. Grid cell averages were calculated on the basis of the latitude and longitude limits between 40° to 60°N and 45° to 100°E, respectively. These values were subsequently averaged over 5 years, consistently with the ice-core data.

## Supplementary Materials

### This PDF file includes:

Supplementary Text Figs. S1 to S8 Tables S1 and S2 References
