# Supplementary material for: Nontarget screening of a Siberian ice core reveals changes in the pre-industrial to industrial organic aerosol composition
Source: Sci Adv. 2025 Jan 24;11(4):eadr1923. doi: 10.1126/sciadv.adr1923 (PMC11758998; doi:10.1126/sciadv.adr1923)
Supplement: Supplementary file 1 — Supplementary Text Figs. S1 to S8 Tables S1 and S2 References [file sciadv.adr1923_sm.pdf]

Science Advances
AAAS

Supplementary Materials for

Nontarget screening of a Siberian ice core reveals changes in the pre-industrial to industrial organic aerosol composition

François Burgay *et al.*

Corresponding author: Margit Schwikowski, margit.schwikowski@psi.ch

Sci. Adv. **11**, eadr1923 (2025)
DOI: 10.1126/sciadv.adr1923

This PDF file includes:

Supplementary Text
Figs. S1 to S8
Tables S1 and S2
References

SI 1 Sample processing and labware cleaning procedure

After being shipped to Paul Scherrer Institut (Switzerland), the ice cores were cut. Ice cutting was performed in a cold room at -20°C using a modified band saw with a stainless-steel blade and a polytetrafluoroethylene (PTFE) tabletop. All surfaces in contact with the core (i.e., the bandsaw, the tabletop, and the saw guide) were cleaned at the beginning of the cutting day and after each core with acetone (Reag. Ph. Eur., VWR Chemicals). The operators wore gloves covered with PE gloves that were changed regularly to avoid sample cross-contamination. To avoid any contamination from the ice-core drilling operations as well as from the ice core handling and storage, the outermost part (≈1 cm) of each ice-core section was removed. The decontaminated inner ice-core parts were used for organic analyses. A section of 2.4 x 2.5 x 50 cm was cut for organic analyses (including bulk water-insoluble and water-soluble organic carbon analysis, and their radiocarbon content). A total number of 53 samples was collected for the NTS analyses of organic tracers from between 26.55- and 62.87-meters depth. The ice samples for organic analyses were stored in pre-cleaned PE jars and they were kept frozen at -20°C until solid phase extraction (SPE).

To avoid any contamination, all the containers used in this investigation were carefully cleaned and decontaminated. In particular, the cut ice-core sections for organic analyses were stored in 2 L PE jars that were previously filled with ultrapure water (UPW, 18 MΩ cm^{-1}) for at least 24 hours, then rinsed with UPW and refilled with UPW for additional 24 hours. This procedure was repeated five times for each PE jar. The jars were then dried overnight under a Class-1000 laminar flow hood. The 50 mL glass vials (Infochroma, AG) used to store the melted ice samples before solid-phase extraction, the 1.5 mL MS-vials (BG Analytics) used for organic analyses and the 1.5 mL tubes (Eppendorf) used for the standard solution preparation were cleaned as described in Burgay et al., *(38)*. Filter punches of 1 cm in diameter (PALLFLEX, Tissuquartz filters 2500QAT-UP) used for collecting the water-insoluble organic carbon and to filter the samples for the NTS analyses were baked at 800°C for 5 hours prior to use.

For major ions analyses, the 50 mL PE tubes were cleaned like the PE jars, but were dried overnight in an oven at 65°C.

SI 2. Organic tracer analysis.

In brief, 48.5±0.9 mL (n = 53) aliquots were collected from a sample for analyses of water-soluble organic carbon after rinsing 300-500 g of ice with UPW, melting it and filtering under clean conditions and helium atmosphere in the extraction setup *(80)*. Aliquots were spiked with 75 µL of 40 ng g^{-1} internal standard (*p*-hydroxybenzoic acid-(phenyl-$^{13}C_6$)) to achieve a concentration of 0.055 ± 0.003 ng g^{-1} and alkalized with 8 µL of NH_4OH (25% in UPW) to pH ≈ 10. The samples were pre-concentrated using Strong Anionic Exchange (MAX, 1 mL, 10 mg bed weight, Waters) solid-phase extraction (SPE) cartridges and disposable PTFE transfer tubes at a flowrate of 1.4 ± 0.3 mL min^{-1}. The cartridges were previously conditioned with 1 mL of methanol followed by 5 mL of UPW, decontaminated using 500 µL of a 0.16 M HCl solution in methanol and exchanged in their counter ion with 500 µL of a 2% formic acid solution in UPW. After the loading step, the cartridges were wrapped in two aluminum foils and stored at -20°C for two months. It was previously demonstrated that this approach provides a higher analytical reproducibility compared to re-freezing the aliquots after their collection in the 50 mL glass vials *(38)*. Before elution, the cartridges were thawed at room temperature under a Class-1000 laminar flowhood for ≈30 min and then eluted using 3x250 µL of a 5% formic acid solution in methanol at a flowrate of 1 mL min^{-1} in pre-cleaned 1.5 mL vials. The 750-µL eluates were pre-concentrated to ≈40 µL at 30°C under a gentle N_2 flow (Reacti-Vap Evaporator, Thermo Fischer Scientific) and then retaken with 475 µL of UPW before analysis. Subsequently, 24 ± 1 µL of 1.4 µg g^{-1} vanillin-(phenyl-$^{13}C_6$) solution (10% v/v MeOH/UPW) was added as an additional internal standard to monitor the instrument performances. The final volume was 530 ± 10 µL, indicating that the samples were enriched by a factor of 92 ± 3.

For analyses, samples were transferred to a thermostated autosampler (T = 10°C) and analyzed within 24 hours with an Ultra-High Performance Liquid Chromatograph (Ultimate 3000, Thermo Fisher Scientific) equipped with an Acclaim™ Organic Acid Column (3 µm, 2.1 x 150 mm, Thermo Fisher Scientific, operated at 50°C) coupled with a High-Resolution Mass Spectrometer (UHPLC-HRMS, Q Exactive Focus, Thermo Fisher Scientific). The injection volume was 20 µL. Chromatographic separation was obtained using a mobile phase consisting of 0.01% formic acid, 1% acetonitrile and 1%

methanol in water (v/v/v, eluent A) and methanol (eluent B) with a flow rate of 0.2 mL min^{-1}, with the following binary elution program: 0-12 min linearly increasing gradient from 8% to 90% of B, 12-15 min isocratic elution at 90% B. Compounds were ionized in a heated electrospray ionization (HESI) source operating in negative mode. Data acquisition was performed in Full-MS mode with a scan range from 70 to 1000 mass-to-charge ratio (*m/z*). Instrumental conditions for electrospray ionization were: sheath gas (N_2) 35 au, auxiliary gas (N_2) 10 au probe heater temperature 300°C, capillary temperature 280 °C, and capillary voltage 2.5 kV. The MS-data were recorded in centroid mode with lock mass at *m/z* 112.98563 (sodium formate cluster). Resolution at *m/z* = 200 was 7E4. Data-dependent MS-MS (dd-MS^2) experiments were also performed with higher-energy collision dissociation (HCD) at 10, 20 and 40 au.

The analysis of the samples was performed over three months (July-September 2022). Method performances were evaluated in terms of mass accuracies, reproducibility of the retention times and internal standard intensities of $^{13}C_6$-p-hydroxybenzoic acid (^{13}C-PHBA), $^{13}C_6$-vanillin (^{13}C-VAN), succinic acid ($C_4H_6O_4$) and pinic acid ($C_9H_{14}O_4$). Mass accuracies ranged between ± 3 ppm, while the variability of the retention times was lower than 0.05 min over the entire analysis period for all the selected compounds. ^{13}C-VAN and ^{13}C-PHBA intensities were used to evaluate the instrumental sensitivity and the solid phase extraction performance, respectively. The observed variability in ^{13}C-VAN intensity was 17.0% RSD. To consider changes in the instrumental sensitivity, data were corrected using the intensity of ^{13}C-VAN. The observed variability in ^{13}C-PHBA intensity was 17.8% RSD (after correction), indicating an overall good reproducibility of the extractions. N=14 procedural UPW blanks were also analyzed to account for any possible contamination during the sample processing and were used in the NTS workflow for background subtraction.

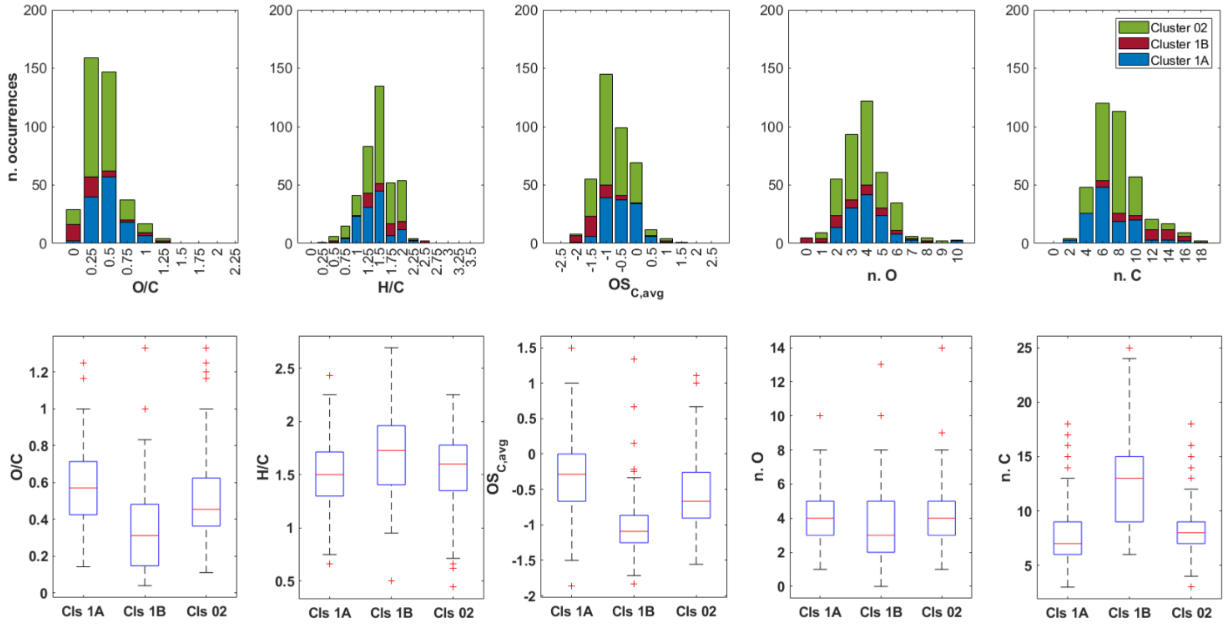

Figure S1 – Distribution of the chemical properties of molecules identified in the Belukha ice core for the different clusters. Distribution (upper panels) and boxplots (lower panels) of oxygen-to-carbon (O/C), hydrogen-to-carbon (H/C) ratios, average carbon oxidation state ($OS_{C,avg}$), number of oxygens (n. O) and number of carbons (n. C) for all the molecules detected in the Belukha ice core and divided by clusters (Cls: see main text for details related to clustering).

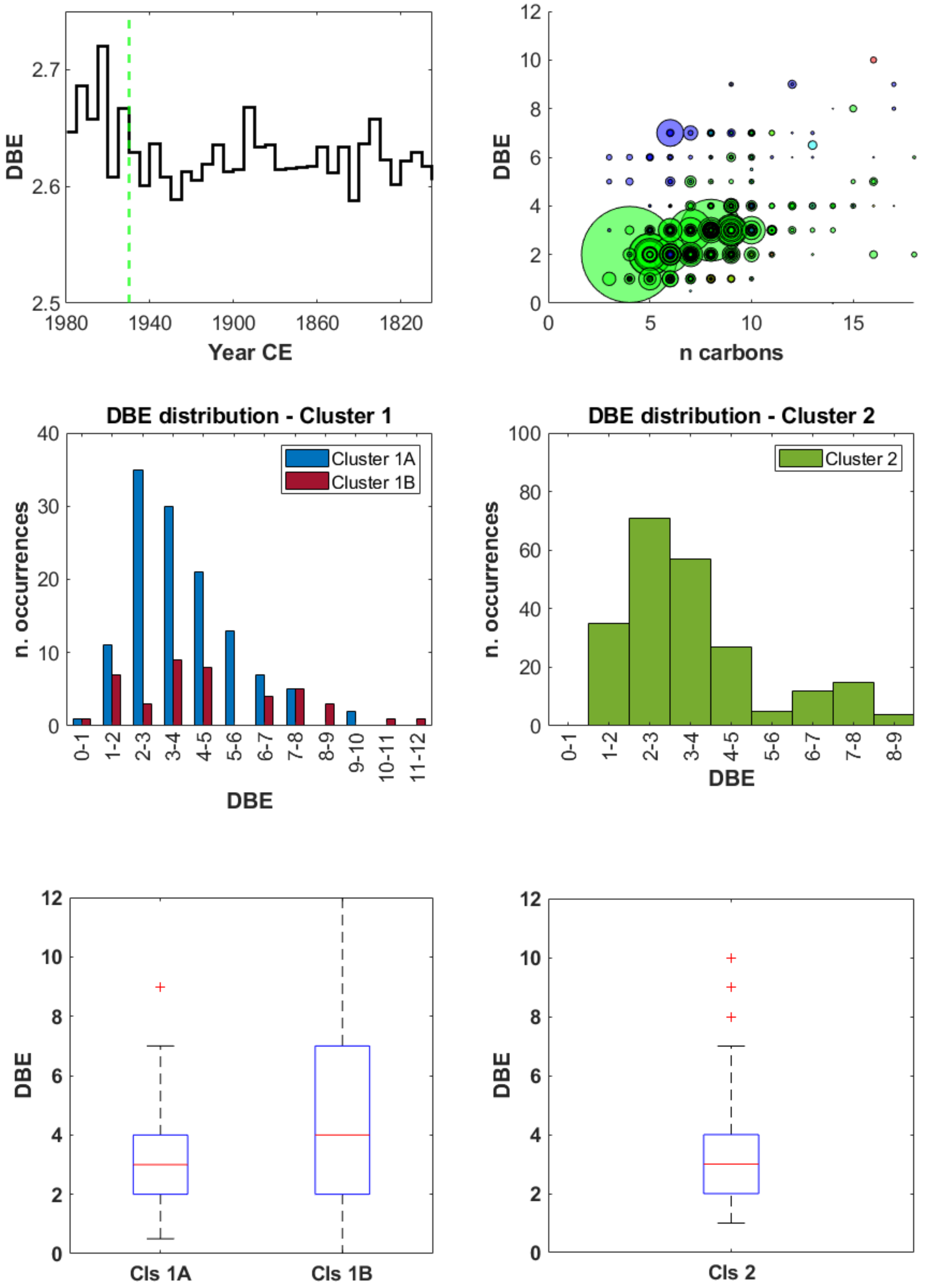

Figure S2– Double Bond Equivalent temporal profile and distribution. Upper panel left: the DBE (Double Bond Equivalent) temporal profile shows an abrupt change point in 1950 CE (dashed vertical green line). Upper panel right: the DBE distribution of the identified compounds. The color of the circles corresponds to the molecular composition of the compounds (green: CHO, blue: CHNO, red: CHOS, yellow: CHNOS, light blue: other), while the size corresponds to the average intensity calculated over the entire record. Middle panel left: DBE distribution for cluster 1. Middle panel right: DBE distribution for cluster 2. Bottom panel left: boxplot showing the DBE distribution for cluster 1. Bottom panel right: boxplot showing the DBE distribution for cluster 2 (see main text for details related to clustering).

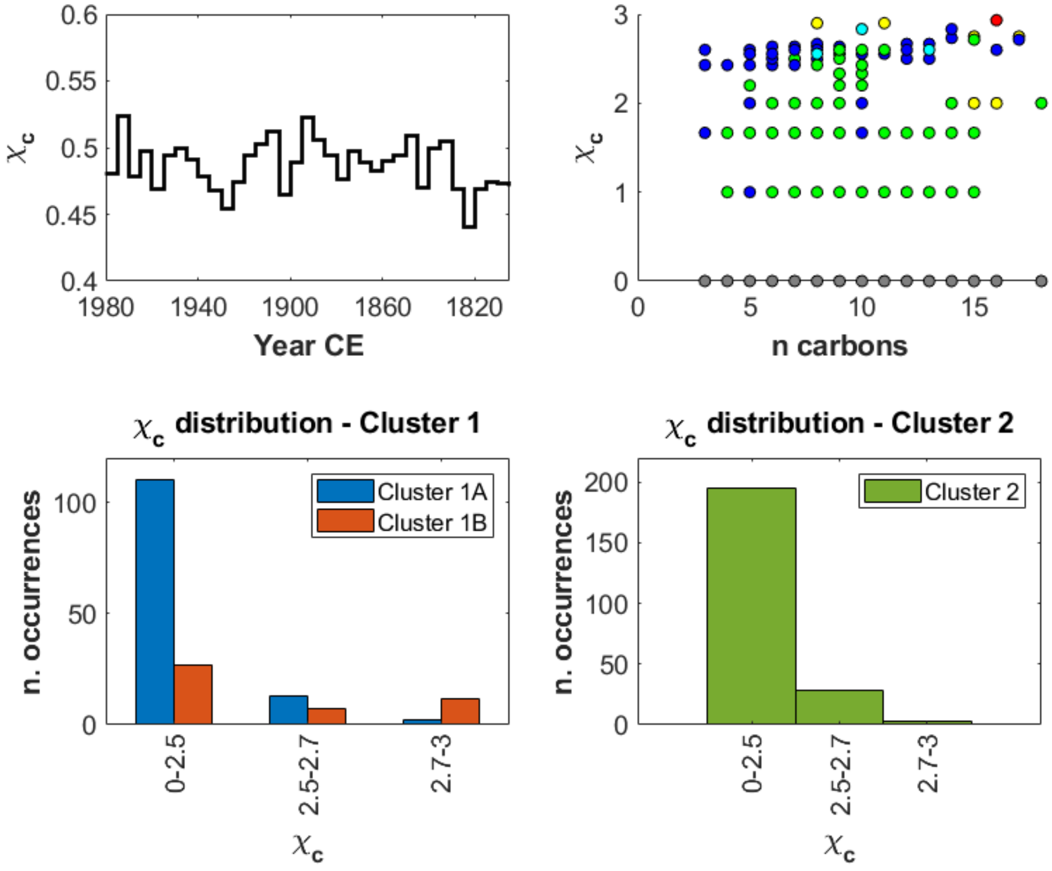

Figure S3– Aromaticity Equivalent (χ_C). Upper panel left: the χ_C temporal profile. Upper panel right: the χ_C distribution of the identified compounds. If there are multiple compounds with the same number of carbon atoms and the same χ_C, they are represented as a single dot. The color of the circles corresponds to the molecular composition of the compounds (green: CHO, blue: CHNO, red: CHOS, yellow: CHNOS, light blue: other). The grey circles represent all the compounds with $\chi_C = 0$. Bottom panel left: χ_C distribution for cluster 1. Bottom panel right: χ_C distribution for cluster 2.

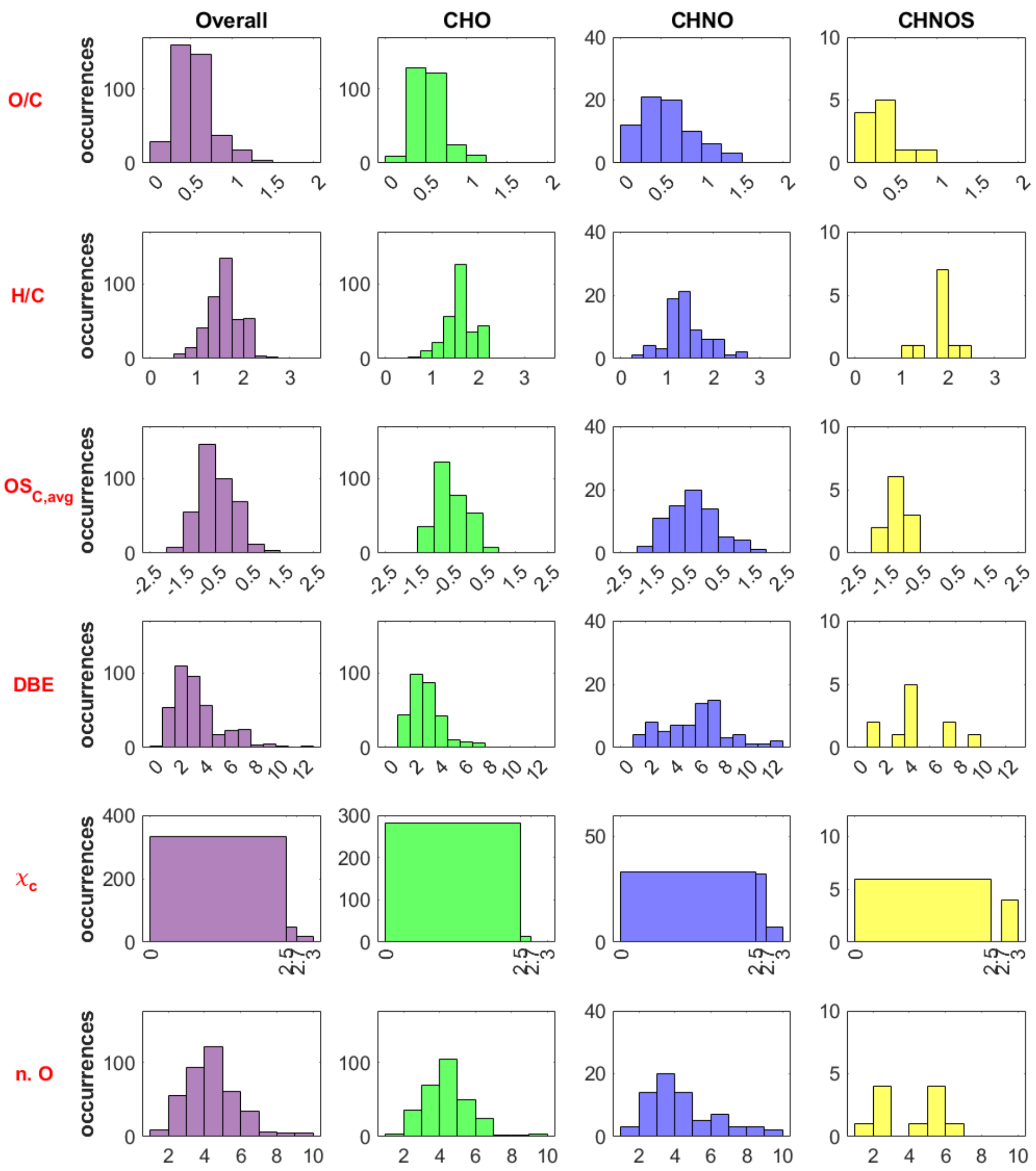

Figure S4 – Distribution of the chemical properties of molecules identified in the Belukha ice core for each molecular class. The oxygen-to-carbon ratio (O/C), hydrogen-to-carbon ratio (H/C), average carbon oxidation state ($OS_{C,avg}$), double bound equivalent (DBE), aromaticity equivalent (χ_c), and number of oxygen (n. O) frequency distributions are shown for different molecular classes.

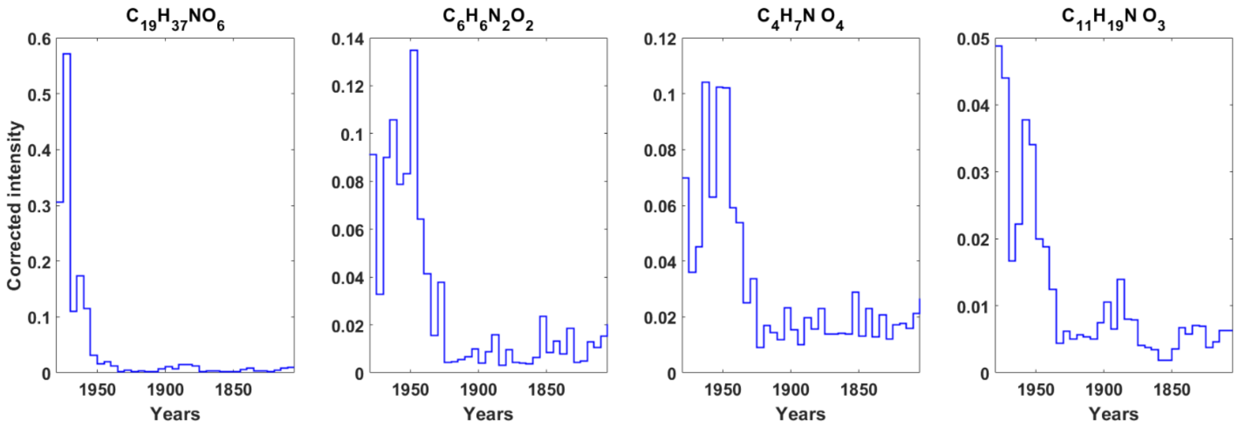

Figure S5 – Selected CHNO profiles. They show a notable increase in the industrial, compared to the pre-industrial period. Note the synchronicity or difference in the time of their emergence.

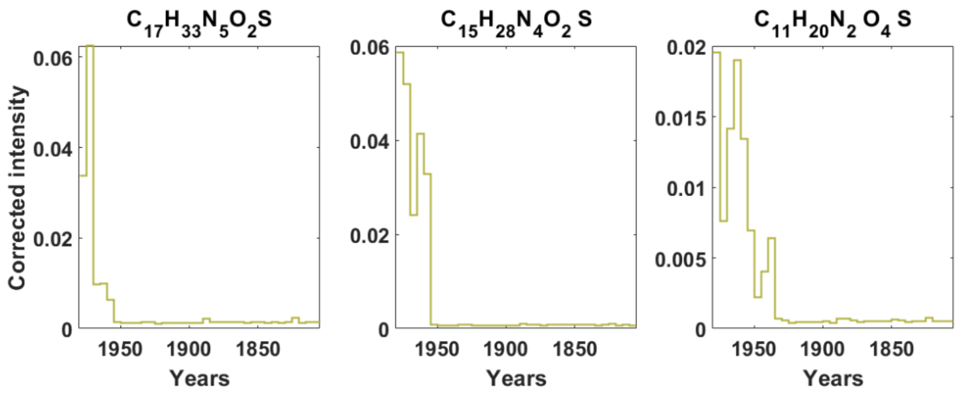

Figure S6 - Selected CHNOS profiles. They show a notable increase in the industrial, compared to the pre-industrial period. Note the synchronicity or difference in the time of their emergence.

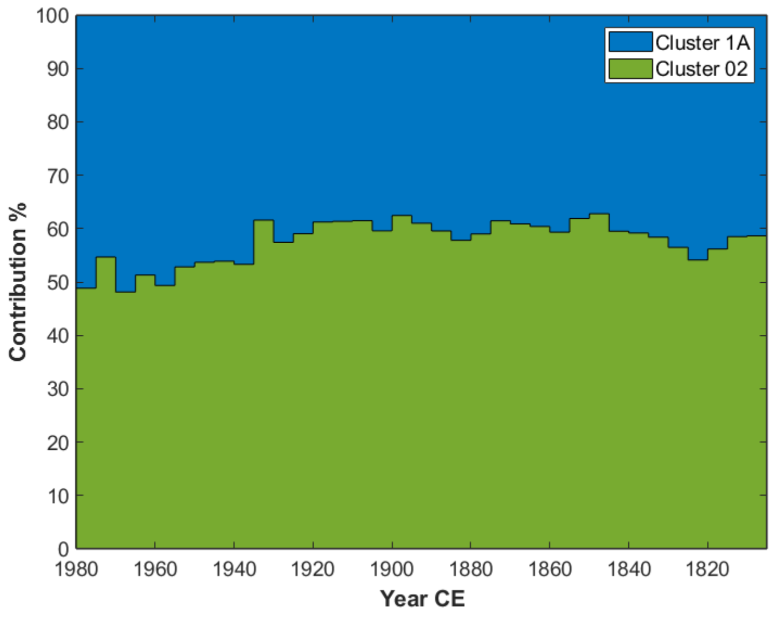

Figure S7 – Relative intensities over time for clusters 1A and 2. In the industrial period a decreasing contribution for cluster 2 is recognizable, while an increasing contribution from cluster 1A can be observed. Cluster 1B was not included.

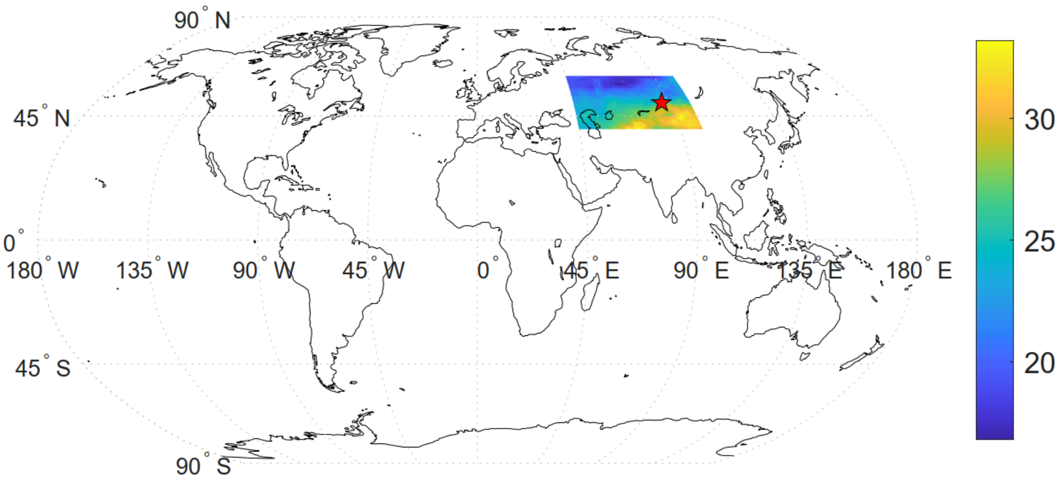

Figure S8 – Source area for the oxidants (OH radicals and O_3). The source area, which is representative of the air masses reaching the Belukha glacier (see main text), is highlighted by the rectangle. For illustrative purposes, plotted values (average mixing ratio in surface layer) refer to O_3 concentration (in ppb) in June 1850. The ice-core location is marked with a red star.

Table S1– Identified compounds. Identified compounds at level 1 (n = 8), level 2 (n = 8) and level 3 (n = 6) using *mzCloud* as MS^2 spectral library. Only compounds with mzCloud Match ≥80 are reported.

**Suspect name**	**Formula**	**RT /min**	**Cluster**	**Identification Level**	**mzCloud Match**
Pimelic acid	$C_7H_{12}O_4$	8.17	2	1	-
Sebacic acid	$C_{10}H_{18}O_4$	11.13	1A	1	-
Glutaric acid	$C_5H_8O_4$	4.44	2	1	-
Adipic acid	$C_6H_{10}O_4$	6.41	1A	1	-
Succinic acid	$C_4H_6O_4$	3.68	1A	1	-
Levulinic acid	$C_5H_8O_3$	3.87	2	1	-
Pinic acid	$C_9H_{14}O_4$	9.03	1A	1	-
p-hydroxybenzoic acid	$C_7H_6O_3$	8.55	1A	1	-
3-Methylsalicylic acid	$C_8H_8O_3$	9.83	1A	3	97.6
3,3 Dimethylglutaric acid	$C_7H_{12}O_4$	8.54	2	2	95.9
4-hydroxy-2-methylbenzoic acid	$C_8H_8O_3$	9.83	1A	3	94.2
4-hydroxyphenylacetic acid	$C_8H_8O_3$	9.83	1A	3	92.9
p-nitrophenol	$C_6H_5NO_3$	10.94	1A	2	92.7

Isobutyric acid	$C_4H_8O_2$	4.41	1A	2	91.5
3-methyladipic acid	$C_7H_{12}O_4$	8.54	2	2	91.2
3-hydroxy-3-methylglutaric acid	$C_6H_{10}O_5$	3.65	2	2	87.2
4-hydroxybenzaldehyde	$C_7H_6O_2$	8.88	2	2	86.9
Cyclohexanecarboxylic acid	$C_7H_{12}O_2$	7.91	2	3	86.3
3-hydroxybutyric acid	$C_4H_8O_3$	3.41	2	3	86.5
Camphanic acid	$C_{10}H_{14}O_4$	8.57	2	2	85.7
Caffeic acid	$C_9H_8O_4$	8.37	2	3	85.4
p-hydroxymethyl-benzoic acid	$C_8H_8O_3$	9.83	1A	3	85.2
4-acetamidobutanoic acid	$C_6H_{11}NO_3$	7.72	2	3	85.2
4-isopropylmalic acid	$C_7H_{12}O_5$	4.31	2	3	84.8
N-Isobutyrylglycine	$C_6H_{11}NO_3$	7.72	2	3	84.3
2-hydroxycinnamic acid	$C_9H_8O_3$	9.20	2	2	83.5
2-hydroxyphenylacetic acid	$C_8H_8O_3$	9.83	1A	3	82.5
4-hydroxyphenylpyruvic acid	$C_9H_8O_4$	8.37	2	3	81.8

2,3-dihydro-1,4-benzodioxine-5-carboxylic acid	$C_9H_8O_4$	8.37	2	3	81.5
Cyclopentylacetic acid	$C_7H_{12}O_2$	7.91	2	3	80.8

Table S2 – Years of abrupt change and correlation coefficients for cluster 1 and other anthropogenic-related species. Cu, Zn, Cd and Sn data are from Eichler et al., 2014 *(32)*. Pb data are from Eichler et al., 2012 *(33)* (* indicates p-value < 0.01).

Element	Source	Year of abrupt change	R
**Cluster 1**	*Atmospheric processing or direct emissions*	1950	1
**Cu**	copper-nickel production (mining, smelting complexes)	1955	**0.82***
**Zn**	Pb-Zn, steel-Fe production	1960	**0.81***
**Cd**	Secondary emission from Pb-Zn and Cu-Ni production	1950	**0.85***
**Sb**	Secondary emission during Cu-Ni and steel-Fe production	1950	**0.85***
**Pb**	road traffic	1950	**0.87***
**SO_4^{2-}**	fossil fuel combustion	1950	**0.72***
**NO_3^-**	high temperature combustion	1965	**0.70***

REFERENCES AND NOTES

1. J. H. Seinfeld, C. Bretherton, K. S. Carslaw, H. Coe, P. J. DeMott, E. J. Dunlea, G. Feingold, S. Ghan, A. B. Guenther, R. Kahn, I. Kraucunas, S. M. Kreidenweis, M. J. Molina, A. Nenes, J. E. Penner, K. A. Prather, V. Ramanathan, V. Ramaswamy, P. J. Rasch, A. R. Ravishankara, D. Rosenfeld, G. Stephens, R. Wood, Improving our fundamental understanding of the role of aerosol−cloud interactions in the climate system. *Proc. Natl. Acad. Sci. U.S.A.* **113**, 5781–5790 (2016).

2. G. McFiggans, P. Artaxo, U. Baltensperger, H. Coe, M. C. Facchini, G. Feingold, S. Fuzzi, M. Gysel, A. Laaksonen, U. Lohmann, T. F. Mentel, D. M. Murphy, C. D. O'Dowd, J. R. Snider, E. Weingartner, The effect of physical and chemical aerosol properties on warm cloud droplet activation. *Atmos. Chem. Phys.* **6**, 2593–2649 (2006).

3. J. L. Jimenez, M. Canagaratna, N. Donahue, A. Prevot, Q. Zhang, J. H. Kroll, P. F. DeCarlo, J. D. Allan, H. Coe, N. L. Ng, A. C. Aiken, K. S. Docherty, I. M. Ulbrich, A. P. Grieshop, A. L. Robinson, J. Duplissy, J. D. Smith, K. R. Wilson, V. A. Lanz, C. Hueglin, Y. L. Sun, J. Tian, A. Laaksonen, T. Raatikainen, J. Rautiainen, P. Vaattovaara, M. Ehn, M. Kulmala, J. M. Tomlinson, D. R. Collins, M. J. Cubison, E. J. Dunlea, J. A. Huffman, T. B. Onasch, M. R. Alfarra, P. I. Williams, K. Bower, Y. Kondo, J. Schneider, F. Drewnick, S. Borrmann, S. Weimer, K. Demerjian, D. Salcedo, L. Cottrell, R. Griffin, A. Takami, T. Miyoshi, S. Hatakeyama, A. Shimono, J. Y. Sun, Y. M. Zhang, K. Dzepina, J. R. Kimmel, D. Sueper, J. T. Jayne, S. C. Herndon, A. M. Trimborn, L. R. Williams, E. C. Wood, A. M. Middlebrook, C. E. Kolb, U. Baltensperger, D. R. Worsnop, Evolution of organic aerosols in the atmosphere. *Science* **326**, 1525–1529 (2009).

4. L. Qi, A. L. Vogel, S. Esmaeilirad, L. Cao, J. Zheng, J.-L. Jaffrezo, P. Fermo, A. Kasper-Giebl, K. R. Daellenbach, M. Chen, X. Ge, U. Baltensperger, A. S. H. Prévôt, J. G. Slowik, A 1-year characterization of organic aerosol composition and sources using an extractive electrospray ionization time-of-flight mass spectrometer (EESI-TOF). *Atmos. Chem. Phys.* **20**, 7875–7893 (2020).

5. J. Cao, S. Situ, Y. Hao, S. Xie, L. Li, Enhanced summertime ozone and SOA from biogenic volatile organic compound (BVOC) emissions due to vegetation biomass variability during 1981–2018 in China. *Atmos. Chem. Phys.* **22**, 2351–2364 (2022).

6. B. C. McDonald, J. A. De Gouw, J. B. Gilman, S. H. Jathar, A. Akherati, C. D. Cappa, J. L. Jimenez, J. Lee-Taylor, P. L. Hayes, S. A. McKeen, Y. Y. Cui, S.-W. Kim, D. R. Gentner, G. Isaacman-Vanwertz, A. H. Goldstein, R. A. Harley, G. J. Frost, J. M. Roberts, T. B. Ryerson, M. Trainer, Volatile chemical products emerging as largest petrochemical source of urban organic emissions. *Science* **359**, 760–764 (2018).

7. M. Hallquist, J. C. Wenger, U. Baltensperger, Y. Rudich, D. Simpson, M. Claeys, J. Dommen, N. Donahue, C. George, A. Goldstein, J. F. Hamilton, H. Herrmann, T. Hoffmann, Y. Iinuma, M. Jang, M. E. Jenkin, J. L. Jimenez, A. Kiendler-Scharr, W. Maenhaut, G. McFiggans, T. F. Mentel, A. Monod, A. S. H. Prévôt, J. H. Seinfeld, J. D. Surratt, R. Szmigielski, J. Wildt, The formation, properties and impact of secondary organic aerosol: Current and emerging issues. *Atmos. Chem. Phys.* **9**, 5155–5236 (2009).

8. A. H. Goldstein, I. E. Galbally, Known and unexplored organic constituent in the Earth's atmosphere. *Environ. Sci. Technol*. **41**, 1514–1521 (2007).

9. V. Lanz, A. Prévôt, M. Alfarra, S. Weimer, C. Mohr, P. DeCarlo, M. Gianini, C. Hueglin, J. Schneider, O. Favez, B. D'Anna, C. George, U. Baltensperger, Characterization of aerosol chemical composition with aerosol mass spectrometry in Central Europe: An overview. *Atmos. Chem. Phys.* **10**, 10453–10471 (2010).

10. J. Ma, F. Ungeheuer, F. Zheng, W. Du, Y. Wang, J. Cai, Y. Zhou, C. Yan, Y. Liu, M. Kulmala, K. R. Daellenbach, A. L. Vogel, Nontarget screening exhibits a seasonal cycle of PM2. 5 organic aerosol composition in Beijing. *Environ. Sci. Technol.* **56**, 7017–7028 (2022).

11. C. Giorio, C. Bortolini, I. Kourtchev, A. Tapparo, S. Bogialli, M. Kalberer, Direct target and non-target analysis of urban aerosol sample extracts using atmospheric pressure photoionisation high-resolution mass spectrometry. *Chemosphere* **224**, 786–795 (2019).

12. K. Wang, R.-J. Huang, M. Brüggemann, Y. Zhang, L. Yang, H. Ni, J. Guo, M. Wang, J. Han, M. Bilde, M. Glasius, T. Hoffmann, Urban organic aerosol composition in eastern China differs from north to south: Molecular insight from a liquid chromatography–mass spectrometry (Orbitrap) study. *Atmos. Chem. Phys.* **21**, 9089–9104 (2021).

13. K. R. Daellenbach, I. Kourtchev, A. L. Vogel, E. A. Bruns, J. Jiang, T. Petäjä, J.-L. Jaffrezo, S. Aksoyoglu, M. Kalberer, U. Baltensperger, I. El Haddad, A. S. H. Prévôt, Impact of anthropogenic and biogenic sources on the seasonal variation in the molecular composition of urban organic aerosols: A field and laboratory study using ultra-high-resolution mass spectrometry. *Atmos. Chem. Phys.* **19**, 5973–5991 (2019).

14. A. L. Vogel, A. Lauer, L. Fang, K. Arturi, F. Bachmeier, K. R. Daellenbach, T. Käser, A. Vlachou, V. Pospisilova, U. Baltensperger, I. El Haddad, M. Schwikowski, S. Bjelić, A comprehensive nontarget analysis for the molecular reconstruction of organic aerosol composition from glacier ice cores. *Environ. Sci. Technol.* **53**, 12565–12575 (2019).

15. A. M. Grannas, W. C. Hockaday, P. G. Hatcher, L. G. Thompson, E. Mosley-Thompson, New revelations on the nature of organic matter in ice cores. *J. Geophys. Res. Atmos.* **111**, 10.1029/2005JD006251 (2006).

16. R. Zangrando, V. Zanella, O. Karroca, E. Barbaro, N. M. Kehrwald, D. Battistel, E. Morabito, A. Gambaro, C. Barbante, Dissolved organic matter in the deep TALDICE ice core: A nano-UPLC-nano-ESI-HRMS method. *Sci. Total Environ.* **700**, 134432 (2020).

17. S. Schüpbach, H. Fischer, M. Bigler, T. Erhardt, G. Gfeller, D. Leuenberger, O. Mini, R. Mulvaney, N. J. Abram, L. Fleet, M. M. Frey, E. Thomas, A. Svensson, D. Dahl-Jensen, E. Kettner, H. Kjaer, I. Seierstad, J. P. Steffensen, S. O. Rasmussen, P. Vallelonga, M. Winstrup, A. Wegner, B. Twarloh, K. Wolff, E. W. Wolff, Greenland records of aerosol source and atmospheric lifetime changes from the Eemian to the Holocene. *Nat. Commun.* **9**, 1476 (2018).

18. E. Wolff, C. Barbante, S. Becagli, M. Bigler, C. Boutron, E. Castellano, M. De Angelis, U. Federer, H. Fischer, F. Fundel, M. Hansson, M. Hutterli, U. Jonsell, T. Karlin, P. Kaufmann, F. Lambert, G. C. Littot, R. Mulvaney, R. Röthlisberger, U. Ruth, A. Wegner, Changes in environment over the last 800,000 years from chemical analysis of the EPICA Dome C ice core. *Quat. Sci. Rev.* **29**, 285–295 (2010).

19. F. Burgay, A. Spolaor, J. Gabrieli, G. Cozzi, C. Turetta, P. Vallelonga, C. Barbante, Atmospheric iron supply and marine productivity in the glacial North Pacific Ocean. *Clim. Past* **17**, 491–505 (2021).

20. T. M. Jenk, S. Szidat, M. Schwikowski, H. W. Gäggeler, S. Brütsch, L. Wacker, H.-A. Synal, M. Saurer, Radiocarbon analysis in an Alpine ice core: Record of anthropogenic and biogenic contributions to carbonaceous aerosols in the past (1650? 1940). *Atmos. Chem. Phys.* **6**, 5381–5390 (2006).

21. M. Legrand, S. Preunkert, S. Kutuzov, G. Siour, V. Mikhalenko, E. Dolgova, R. Friedrich, 20th Century changes of DOC and its ^{14}C signature archived in Caucasus ice-core: Implications for past sources of organic carbon aerosol in south-eastern Europe. *J. Geophys. Res. Atmos.* **129**, 2023JD040121 (2024).

22. C. Müller-Tautges, A. Eichler, M. Schwikowski, G. Pezzatti, M. Conedera, T. Hoffmann, Historic records of organic compounds from a high Alpine glacier: Influences of biomass burning, anthropogenic emissions, and dust transport. *Atmos. Chem. Phys.* **16**, 1029–1043 (2016).

23. P. Zennaro, N. Kehrwald, J. R. McConnell, S. Schüpbach, O. J. Maselli, J. Marlon, P. Vallelonga, D. Leuenberger, R. Zangrando, A. Spolaor, M. Borrotti, E. Barbaro, A. Gambaro, C. Barbante, Fire in ice: Two millennia of boreal forest fire history from the Greenland NEEM ice core. *Clim. Past* **10**, 1905–1924 (2014).

24. M. Vecchiato, A. Gambaro, N. M. Kehrwald, P. Ginot, S. Kutuzov, V. Mikhalenko, C. Barbante, The Great acceleration of fragrances and PAHs archived in an ice core from Elbrus, Caucasus. *Sci. Rep.* **10**, 10661 (2020).

25. W. F. Hartz, M. K. Björnsdotter, L. W. Yeung, A. Hodson, E. R. Thomas, J. D. Humby, C. Day, I. E. Jogsten, A. Kärrman, R. Kallenborn, Levels and distribution profiles of per-and polyfluoroalkyl substances (PFAS) in a high Arctic Svalbard ice core. *Sci. Total Environ.* **871**, 161830 (2023).

26. A. C. F. King, E. R. Thomas, J. B. Pedro, B. Markle, M. Potocki, S. L. Jackson, E. Wolff, M. Kalberer, Organic compounds in a sub-Antarctic ice core: A potential suite of sea ice markers. *Geophys. Res. Lett.* **46**, 9930–9939 (2019).

27. B. Alexander, L. J. Mickley, Paleo-perspectives on potential future changes in the oxidative capacity of the atmosphere due to climate change and anthropogenic emissions. *Curr. Pollut. Rep.* **1**, 57–69 (2015).

28. V. Naik, A. Voulgarakis, A. M. Fiore, L. W. Horowitz, J.-F. Lamarque, M. Lin, M. J. Prather, P. Young, D. Bergmann, P. Cameron-Smith, I. Cionni, W. J. Collins, S. B. Dalsøren, R. Doherty, V. Eyring, G. Faluvegi, G. A. Folberth, B. Josse, Y. H. Lee, I. A. M. Kenzie, T. Nagashima, T. P. C. van Noije, D. A. Plummer, M. Righi, S. T. Rumbold, R. Skeie, D. T. Shindell, D. S. Stevenson, S. Strode, K. Sudo, S. Szopa, G. Zeng, Preindustrial to present-day changes in tropospheric hydroxyl radical and methane lifetime from the atmospheric chemistry and climate model intercomparison project (ACCMIP). *Atmos. Chem. Phys.* **13**, 5277–5298 (2013).

29. L. T. Murray, L. J. Mickley, J. O. Kaplan, E. D. Sofen, M. Pfeiffer, B. Alexander, Factors controlling variability in the oxidative capacity of the troposphere since the Last Glacial Maximum. *Atmos. Chem. Phys.* **14**, 3589–3622 (2014).

30. A. Sigg, A. Neftel, Evidence for a 50% increase in H_20_2 over the past 200 years from a Greenland ice core. *Nature* **351**, 557–559 (1991).

31. T. Staffelbach, A. Neftel, B. Stauffer, D. Jacob, A record of the atmospheric methane sink from formaldehyde in polar ice cores. *Nature* **349**, 603–605 (1991).

32. A. Eichler, L. Tobler, S. Eyrikh, N. Malygina, T. Papina, M. Schwikowski, Ice-core based assessment of historical anthropogenic heavy metal (Cd, Cu, Sb, Zn) emissions in the Soviet Union. *Environ. Sci. Technol.* **48**, 2635–2642 (2014).

33. A. Eichler, L. Tobler, S. Eyrikh, G. Gramlich, N. Malygina, T. Papina, M. Schwikowski, Three centuries of Eastern European and Altai lead emissions recorded in a Belukha ice core. *Environ. Sci. Technol.* **46**, 4323–4330 (2012).

34. S. Eyrikh, A. Eichler, L. Tobler, N. Malygina, T. Papina, M. Schwikowski, A 320 year ice-core record of atmospheric Hg pollution in the Altai, Central Asia. *Environ. Sci. Technol.* **51**, 11597–11606 (2017).

35. A. Eichler, W. Tinner, S. Brütsch, S. Olivier, T. Papina, M. Schwikowski, An ice-core based history of Siberian forest fires since AD 1250. *Quat. Sci. Rev.* **30**, 1027–1034 (2011).

36. T. Papina, T. Blyakharchuk, A. Eichler, N. Malygina, E. Mitrofanova, M. Schwikowski, Biological proxies recorded in a Belukha ice core, Russian Altai. *Clim. Past* **9**, 2399–2411 (2013).

37. A. Eichler, S. Brütsch, S. Olivier, T. Papina, M. Schwikowski, A 750 year ice core record of past biogenic emissions from Siberian boreal forests. *Geophys. Res. Lett.* **36**, 10.1029/2009GL038807 (2009).

38. F. Burgay, D. Salionov, C. J. Huber, T. Singer, A. Eichler, F. Ungeheuer, A. Vogel, M. Schwikowski, S. Bjelić, Hybrid targeted/untargeted screening method for the determination of wildfire and water-soluble organic tracers in ice cores and snow. *Anal. Chem.* **95**, 11456–11466 (2023).

39. F. Ungeheuer, D. van Pinxteren, A. L. Vogel, Identification and source attribution of organic compounds in ultrafine particles near Frankfurt International Airport. *Atmos. Chem. Phys.* **21**, 3763–3775 (2021).

40. M. M. Yassine, M. Harir, E. Dabek-Zlotorzynska, P. Schmitt-Kopplin, Structural characterization of organic aerosol using Fourier transform ion cyclotron resonance mass spectrometry: Aromaticity equivalent approach. *Rapid Commun. Mass Spectrom.* **28**, 2445–2454 (2014).

41. D. Thomsen, L. D. Thomsen, E. M. Iversen, T. N. Björgvinsdóttir, S. F. Vinther, J. T. Skønager, T. Hoffmann, J. Elm, M. Bilde, M. Glasius, Ozonolysis of α-pinene and Δ3-carene mixtures: Formation of dimers with two precursors. *Environ. Sci. Technol.* **56**, 16643–16651 (2022).

42. F. Mahrt, L. Peng, J. Zaks, Y. Huang, P. E. Ohno, N. R. Smith, F. K. Gregson, Y. Qin, C. L. Faiola, S. T. Martin, S. A. Nizkorodov, M. Ammann, A. K. Bertram, Not all types of secondary organic aerosol mix: Two phases observed when mixing different secondary organic aerosol types. *Atmos. Chem. Phys.* **22**, 13783–13796 (2022).

43. J. H. Kroll, N. M. Donahue, J. L. Jimenez, S. H. Kessler, M. R. Canagaratna, K. R. Wilson, K. E. Altieri, L. R. Mazzoleni, A. S. Wozniak, H. Bluhm, E. R. Mysak, J. D. Smith, C. E. Kolb, D. R. Worsnop, Carbon oxidation state as a metric for describing the chemistry of atmospheric organic aerosol. *Nat. Chem.* **3**, 133–139 (2011).

44. M. Sueur, J. F. Maillard, O. Lacroix-Andrivet, C. P. Rüger, P. Giusti, H. Lavanant, C. Afonso, PyC2MC: An open-source software solution for visualization and treatment of high-resolution mass spectrometry data. *J. Am. Soc. Mass Spectrom.* **34**, 617–626 (2023).

45. A. P. Praplan, S. Schobesberger, F. Bianchi, M. P. Rissanen, M. Ehn, T. Jokinen, H. Junninen, A. Adamov, A. Amorim, J. Dommen, J. Duplissy, J. Hakala, A. Hansel, M. Heinritzi, J. Kangasluoma, J. Kirkby, M. Krapf, A. Kürten, K. Lehtipalo, F. Riccobono, L. Rondo, N. Sarnela, M. Simon, A. Tomé, J. Tröstl, P. M. Winkler, C. Williamson, P. Ye, J. Curtius, U. Baltensperger, N. M. Donahue, M. Kulmala, D. R. Worsnop, Elemental composition and clustering behaviour of α-pinene oxidation products for different oxidation conditions. *Atmos. Chem. Phys.* **15**, 4145–4159 (2015).

46. E. L. Schymanski, J. Jeon, R. Gulde, K. Fenner, M. Ruff, H. P. Singer, J. Hollender, Identifying small molecules via high resolution mass spectrometry: Communicating confidence. *Environ. Sci. Technol.* **48**, 2097–2098 (2014).

47. B. R. T. Simoneit, Biomass burning—A review of organic tracers for smoke from incomplete combustion. *Appl. Geochem.* **17**, 129–162 (2002).

48. J. Cain, A. Laskin, M. R. Kholghy, M. J. Thomson, H. Wang, Molecular characterization of organic content of soot along the centerline of a coflow diffusion flame. *Phys. Chem. Chem. Phys.* **16**, 25862–25875 (2014).

49. H. Wang, Y. Gao, S. Wang, X. Wu, Y. Liu, X. Li, D. Huang, S. Lou, Z. Wu, S. Guo, S. Jing, Y. Li, C. Huang, G. S. Tyndall, J. J. Orlando, X. Zhang, Atmospheric processing of nitrophenols and nitrocresols from biomass burning emissions. *J. Geophys. Res. Atmos.* **125**, e2020JD033401 (2020).

50. Y. Shao, A. Voliotis, M. Du, Y. Wang, K. Pereira, J. Hamilton, M. R. Alfarra, G. McFiggans, Chemical composition of secondary organic aerosol particles formed from mixtures of anthropogenic and biogenic precursors. *Atmos. Chem. Phys.* **22**, 9799–9826 (2022).

51. C. Mohr, F. D. Lopez-Hilfiker, P. Zotter, A. S. Prévôt, L. Xu, N. L. Ng, S. C. Herndon, L. R. Williams, J. P. Franklin, M. S. Zahniser, D. R. Worsnop, W. B. Knighton, A. C. Aiken, K. J. Gorkowski, M. K. Dubey, J. D. Allan, J. A. Thornton, Contribution of nitrated phenols to wood burning brown carbon light absorption in Detling, United Kingdom during winter time. *Environ. Sci. Technol.* **47**, 6316–6324 (2013).

52. D. Leppla, "Comprehensive study of secondary organic aerosol particles from the Amazon Rainforest by high-resolution mass spectrometry", thesis, Johannes Gutenberg-Universität Mainz (2021).

53. C. Zuth, A. L. Vogel, S. Ockenfeld, R. Huesmann, T. Hoffmann, Ultrahigh-resolution mass spectrometry in real time: Atmospheric pressure chemical ionization Orbitrap mass spectrometry of atmospheric organic aerosol. *Anal. Chem.* **90**, 8816–8823 (2018).

54. L. Qi, M. Chen, G. Stefenelli, V. Pospisilova, Y. Tong, A. Bertrand, C. Hueglin, X. Ge, U. Baltensperger, A. S. Prévôt, J. G. Slowik, Organic aerosol source apportionment in Zurich using an extractive electrospray ionization time-of-flight mass spectrometer (EESI-TOF-MS)–Part 2: Biomass burning influences in winter. *Atmos. Chem. and Phys.* **19**, 8037–8062 (2019).

55. D. Bhattu, S. N. Tripathi, H. S. Bhowmik, V. Moschos, C. P. Lee, M. Rauber, G. Salazar, G. Abbaszade, T. Cui, J. G. Slowik, P. Vats, S. Mishra, V. Lalchandani, R. Satish, P. Rai, R. Casotto, A. Tobler, V. Kumar, Y. Hao, L. Qi, P. Khare, M. I. Manousakas, Q. Wang, Y. Han, J. Tian, S. Darfeuil, M. C. Minguillon, C. Hueglin, S. Conil, N. Rastogi, A. K. Srivastava, D. Ganguly, S. Bjelic, F. Canonaco, J. Schnelle-Kreis, P. A. Dominutti, J.-L. Jaffrezo, S. Szidat,

Y. Chen, J. Cao, U. Baltensperger, G. Uzu, K. R. Daellenbach, I. El Haddad, A. S. H. Prévôt, Local incomplete combustion emissions define the PM2. 5 oxidative potential in Northern India. *Nat. Commun.* **15**, 3517 (2024).

56. A. Eichler, M. Legrand, T. M. Jenk, S. Preunkert, C. Andersson, S. Eckhardt, M. Engardt, A. Plach, M. Schwikowski, Consistent histories of anthropogenic western European air pollution preserved in different Alpine ice cores. *Cryosphere* **17**, 2119–2137 (2023).

57. J. Ray, S. R. McDow, Dicarboxylic acid concentration trends and sampling artifacts. *Atmos. Environ.* **39**, 7906–7919 (2005).

58. E. Aruffo, J. Wang, J. Ye, P. Ohno, Y. Qin, M. Stewart, K. McKinney, P. Di Carlo, S. T. Martin, Partitioning of organonitrates in the production of secondary organic aerosols from α-pinene photo-oxidation. *Environ. Sci. Technol.* **56**, 5421–5429 (2022).

59. A. Zare, P. S. Romer, T. Nguyen, F. N. Keutsch, K. Skog, R. C. Cohen, A comprehensive organic nitrate chemistry: Insights into the lifetime of atmospheric organic nitrates. *Atmos. Chem. Phys.* **18**, 15419–15436 (2018).

60. P. J. Ziemann, R. Atkinson, Kinetics, products, and mechanisms of secondary organic aerosol formation. *Chem. Soc. Rev.* **41**, 6582–6605 (2012).

61. T. Nah, J. Sanchez, C. M. Boyd, N. L. Ng, Photochemical aging of α-pinene and β-pinene secondary organic aerosol formed from nitrate radical oxidation. *Environ. Sci. Technol.* **50**, 222–231 (2016).

62. J. Lelieveld, F. Dentener, W. Peters, M. Krol, On the role of hydroxyl radicals in the self-cleansing capacity of the troposphere. *Atmos. Chem. Phys.* **4**, 2337–2344 (2004).

63. J. H. Kroll, J. H. Seinfeld, Chemistry of secondary organic aerosol: Formation and evolution of low-volatility organics in the atmosphere. *Atmos. Environ.* **42**, 3593–3624 (2008).

64. R. M. Hoesly, S. J. Smith, L. Feng, Z. Klimont, G. Janssens-Maenhout, T. Pitkanen, J. J. Seibert, L. Vu, R. J. Andres, R. M. Bolt, T. C. Bond, L. Dawidowski, N. Kholod, J.-i. Kurokawa, M. Li, L. Liu, Z. Lu, M. C. P. Moura, P. R. O'Rourke, Q. Zhang, Historical

(1750–2014) anthropogenic emissions of reactive gases and aerosols from the Community Emissions Data System (CEDS). *Geosci. Model Dev.* **11**, 369–408 (2018).

65. C. Gao, A. Robock, S. Self, J. B. Witter, J. P. Steffenson, H. B. Clausen, M. L. Siggaard-Andersen, S. Johnsen, P. A. Mayewski, C. Ammann, The 1452 or 1453 AD Kuwae eruption signal derived from multiple ice core records: Greatest volcanic sulfate event of the past 700 years. *J. Geophys. Res. Atmos.* **111**, (2006).

66. J. Cole-Dai, D. Ferris, A. Lanciki, J. Savarino, M. Baroni, M. H. Thiemens, Cold decade (AD 1810–1819) caused by Tambora (1815) and another (1809) stratospheric volcanic eruption. *Geophys. Res. Lett.* **36**, 10.1029/2009GL040882 (2009).

67. J. D. Surratt, M. Lewandowski, J. H. Offenberg, M. Jaoui, T. E. Kleindienst, E. O. Edney, J. H. Seinfeld, Effect of acidity on secondary organic aerosol formation from isoprene. *Environ. Sci. Technol.* **41**, 5363–5369 (2007).

68. S. Gao, J. J. Camarero, F. Babst, E. Liang, Global tree growth resilience to cold extremes following the Tambora volcanic eruption. *Nat. Commun.* **14**, 6616 (2023).

69. L. S. Glaze, S. M. Baloga, L. Wilson, Transport of atmospheric water vapor by volcanic eruption columns. *J. Geophys. Res. Atmos.* **102**, 6099–6108 (1997).

70. O. Garmash, M. H. Hermanson, E. Isaksson, M. Schwikowski, D. Divine, C. Teixeira, D. C. G. Muir, Deposition history of polychlorinated biphenyls to the Lomonosovfonna glacier, Svalbard: A 209 congener analysis. *Environ. Sci. Technol.* **47**, 12064–12072 (2013).

71. J. Gabrieli, P. Vallelonga, G. Cozzi, P. Gabrielli, A. Gambaro, M. Sigl, F. Decet, M. Schwikowski, H. Gäggeler, C. Boutron, P. Cescon, C. Barbante, Post 17th-century changes of European PAH emissions recorded in high-altitude alpine snow and ice. *Environ. Sci. Technol.* **44**, 3260–3266 (2010).

72. M. Brüggemann, R. Xu, A. Tilgner, K. C. Kwong, A. Mutzel, H. Y. Poon, T. Otto, T. Schaefer, L. Poulain, M. N. Chan, H. Herrmsann, Organosulfates in ambient aerosol: State of

knowledge and future research directions on formation, abundance, fate, and importance. *Environ. Sci. Technol.* **54**, 3767–3782 (2020).

73. H. Müller, J. T. Andersson, W. Schrader, Characterization of high-molecular-weight sulfur-containing aromatics in vacuum residues using Fourier transform ion cyclotron resonance mass spectrometry. *Anal. Chem.* **77**, 2536–2543 (2005).

74. X. Chen, H. Li, L. Zhang, Q. Shi, S. Zhao, C. Xu, Direct sulfur-containing compounds analysis in petroleum via (+) ESI FT-ICR MS using HBF_4 as ionization promoter. *Fuel* **278**, 118334 (2020).

75. S. Olivier, M. Schwikowski, S. Brütsch, S. Eyrikh, H. W. Gäggeler, M. Lüthi, T. Papina, M. Saurer, U. Schotterer, L. Tobler, E. Vogel, Glaciochemical investigation of an ice core from Belukha glacier, Siberian Altai. *Geophys. Res. Lett.* **30**, 10.1029/2003GL018290 (2003).

76. V. Dulio, B. van Bavel, E. Brorström-Lundén, J. Harmsen, J. Hollender, M. Schlabach, J. Slobodnik, K. Thomas, J. Koschorreck, Emerging pollutants in the EU: 10 years of NORMAN in support of environmental policies and regulations. *Environ. Sci. Eur.* **30**, 5 (2018).

77. S. E. Avak, J. C. Trachsel, J. Edebeli, S. Brütsch, T. Bartels-Rausch, M. Schneebeli, M. Schwikowski, A. Eichler, Melt-induced fractionation of major ions and trace elements in an Alpine snowpack. *J. Geophys. Res. Earth* **124**, 1647–1657 (2019).

78. K. B. Rodgers, S.-S. Lee, N. Rosenbloom, A. Timmermann, G. Danabasoglu, C. Deser, J. Edwards, J.-E. Kim, I. R. Simpson, K. Stein, M. F. Stuecker, R. Yamaguchi, T. Bódai, E.-S. Chung, L. Huang, W. M. Kim, J.-F. Lamarque, D. L. Lombardozzi, W. R. Wieder, S. G. Yeager, Ubiquity of human-induced changes in climate variability. *Earth Syst. Dynam.* **12**, 1393–1411 (2021).

79. C. A. Greene, K. Thirumalai, K. A. Kearney, J. M. Delgado, W. Schwanghart, N. S. Wolfenbarger, K. M. Thyng, D. E. Gwyther, A. S. Gardner, D. D. Blankenship, The climate data toolbox for MATLAB. *Geochem. Geophys. Geosyst.* **20**, 3774–3781 (2019).

80. L. Fang, J. Schindler, T. Jenk, C. Uglietti, S. Szidat, M. Schwikowski, Extraction of dissolved organic carbon from glacier ice for radiocarbon analysis. *Radiocarbon* **61**, 681–694 (2019).
